# Diabetes and blood glucose monitoring knowledge and practices among pharmacy professionals in Cambodia and Viet Nam: digital survey and education

**DOI:** 10.1186/s12909-023-04449-0

**Published:** 2023-06-29

**Authors:** Cathy Haldane, Josselyn Neukom, Jaca Maison Lailo, Kol Hero, Beatrice Vetter

**Affiliations:** 1 FIND, Geneva, Switzerland; 2 SwipeRx, Singapore, Singapore; 3 Department of Preventive Medicine, Ministry of Health, Phnom Penh, Cambodia

**Keywords:** Diabetes mellitus, Blood glucose self-monitoring, Continuing education, Pharmacists, Southeastern Asia, Digital health, Developing countries

## Abstract

**Background:**

In Southeast Asia, pharmacies are critical sources of healthcare advice for under-served communities, including those with/at risk of diabetes.

**Aim:**

Explore knowledge/practices relating to diabetes and blood glucose monitoring (BGM) among pharmacy professionals in Cambodia and Viet Nam, using digital professional education to address gaps.

**Methods:**

An online survey was distributed to pharmacy professionals in Cambodia and Viet Nam registered on SwipeRx mobile application. Eligible participants dispensed medicines and/or were involved in purchasing products, and worked at retail pharmacies stocking ≥ 1 BGM product. An accredited continuing professional development module was then made available to pharmacy professionals and students on SwipeRx in both countries. After completing the 1–2 h module, users were required to correctly answer ≥ 60% (Cambodia) or ≥ 70% (Viet Nam) of knowledge assessment questions to achieve accreditation units from local partners.

**Results:**

Whereas 33% of survey respondents in Cambodia (N = 386) and 63% in Viet Nam (N = 375) reported performing blood glucose testing at the pharmacy, only 19% and 14% were aware that clients taking multiple daily doses of insulin should check blood glucose levels several times a day. Of 1,137 and 399 pharmacy professionals/students who completed the module and passed the assessment in Cambodia and Viet Nam, 1,124 (99%) and 376 (94%) received accreditation. Knowledge levels improved substantially in 10 of 14 learning areas in Cambodia and 6 of 10 in Viet Nam.

**Conclusions:**

Digital education can strengthen pharmacy professional capacity to provide comprehensive and accurate information on diabetes management and the awareness of quality BGM products in Southeast Asia.

**Supplementary Information:**

The online version contains supplementary material available at 10.1186/s12909-023-04449-0.

## Background

An estimated 90.2 million adults 20–79 years of age in Southeast Asia and 205.6 million in the Western Pacific region are living with diabetes, figures that are projected to increase to 151.5 and 260.2 million, respectively, by 2045 [1]. Both Cambodia and Viet Nam have a high burden: almost 596,000 adults in Cambodia and 4 million in Viet Nam have diabetes, resulting in an age-adjusted prevalence of 7.3% and 6.1% [1]. The majority of these cases are type 2 diabetes, and around half remain undiagnosed [1]. A higher prevalence of diabetes in urban areas compared with rural areas in Asian and Pacific countries, including Cambodia and Viet Nam, has been reported, although this difference may be narrowing [2].

Poorly managed or undiagnosed diabetes leads to serious and permanent complications, including cardiovascular disease, blindness, kidney failure and lower limb amputation, as well as early death [1]. In the Western Pacific, diabetes was responsible for 2.3 million deaths in 2021 [1]. To reduce the risk of complications, people living with diabetes are advised to follow self-management practices, including monitoring of their blood glucose levels [3]. However, access to quality, convenient and affordable diabetes blood glucose monitoring (BGM) supplies and other diabetes care products in the Asia-Pacific region is limited [4].

Pharmacists are the third largest group of healthcare professionals worldwide [5], and are a preferred source of healthcare advice for under-served communities due to their geographic proximity, convenient opening hours, and low waiting times [6, 7]. In Southeast Asia, pharmacies are already serving communities at risk of diabetes [8], and should be a vital link in community-based programmes for diabetes care. Both non-pharmacological and pharmacological interventions delivered by pharmacists have been shown to result in better glycaemic outcomes in people living with diabetes in low- and middle-income countries [9].

The continuing professional development (CPD) model has been shown to support the maintenance of up-to-date knowledge and skills among pharmacists in low- and middle-income countries [10], ensuring competence and optimal patient care. Accredited CPD modules designed for pharmacists focussing on diabetes and BGM could be a key opportunity to improve knowledge and practices to improve access to diabetes testing, treatment and lifestyle counselling in Southeast Asia. Effective CPD must address existing gaps in pharmacists’ knowledge and practices; however, data on pharmacists’ understanding of diabetes and their current diabetes-related practices in Southeast Asia are currently limited.

This study had two parts: (1) a descriptive, cross-sectional survey to explore knowledge, attitudes, and practices relating to diabetes and BGM among pharmacy professionals in Cambodia and Viet Nam, and to understand access to BGM products; (2) implementation and evaluation of an accredited digital CPD module to address knowledge gaps in both countries.

## Methods

### Survey design and inclusion criteria

An online survey was distributed to registered users of the SwipeRx mobile application platform (https://swiperxapp.com; SwipeRx, formerly known as mClinica, Singapore) in Cambodia and Viet Nam. SwipeRx is a free, all-in-one application that provides various features including continuing professional development modules, pharmaceutical news, discussion boards, adverse drug reporting and drug directories. SwipeRx was selected for this study as it hosts the largest digital network of pharmacy professionals in Southeast Asia (N = 3,930 in Cambodia and N = 25,057 in Viet Nam at the time of this study). Cambodia and Viet Nam were selected from the six countries in the SwipeRx network based on number of users, diabetes burden and association support for digital professional education.

Pharmacy professionals eligible for the survey included pharmacists, pharmacy assistants, pharmacy managers, and pharmacy owners who were registered on SwipeRx, were ≥ 18 years of age, were resident in Cambodia or Viet Nam, and were involved in dispensing medicines and health products to pharmacy clients and/or purchasing products for the pharmacy. In addition, only pharmacy professionals who reported working at a retail pharmacy stocking one or more BGM-related product were eligible to participate. Pharmacy students were excluded from the survey.

### Survey development and distribution

The survey was designed by SwipeRx, formerly known as mClinica, and reviewed by FIND (Geneva, Switzerland). The survey was translated from English into Khmer for Cambodian respondents, and into Vietnamese for those from Viet Nam. The survey was piloted with five pharmacy professionals from three rural and two urban locations in Cambodia and five pharmacy professionals in Viet Nam prior to the study to confirm comprehension and feasibility, and was refined accordingly.

The final survey consisted of two screening questions, to ensure that only pharmacy professionals involved in dispensing medication to pharmacy clients and/or ordering or purchasing products for their pharmacy could access the remaining survey questions. This was followed by 42 questions covering the themes of demographics, client volumes, and diabetes and BGM product knowledge, attitudes, and practices (Supplementary File 1). Respondents could withdraw from the survey at any point.

The survey was hosted on the Qualtrics platform (http://www.qualtrics.com; Qualtrics, Utah, USA) and was distributed via digital invitation containing a weblink to the site through SwipeRx. Pharmacy professionals who followed the link were asked to provide informed consent prior to completing the survey online. Those who did not provide consent could not access the survey questions. Access from the same IP address or phone number was limited to ensure that only one survey response from each respondent was recorded; however, there was no restriction on the number of respondents from each individual pharmacy.

#### Ethics approval

was obtained from the National Ethics Committee for Health Research in Cambodia (approval number 120) and the Center for Creative Initiatives in Health and Population in Viet Nam (approval number 24,052,021/HDPB-CCIHP). All survey respondents provided informed consent.

The survey was available between 9 July and 26 August 2021 in Cambodia and 9 June and 21 July 2021 in Viet Nam. Pharmacy professionals were prompted to complete the online survey via in-app pushes, SMS, email, and SwipeRx social media page posts. It was estimated that the survey would take approximately 15–20 min to complete. Survey respondents who completed a minimum proportion of the survey questions (80%) received a modest incentive in the form of telephone credit equivalent to $5 US dollars. To maintain anonymity, those eligible for the incentive were only asked to share the telephone numbers that they wished the credit to be transferred to. Each respondent was identified by their unique numeric SwipeRx user identifier only.

### Survey sample size

The sample size calculation was calculated based on the following formula, where N was the population of pharmacy professionals registered on SwipeRx in each country at the time of the study. The sample proportion (p) was set at 0.5 to give the most conservative sample size, the margin of error was set at 5%, and the Z statistic was set at 1.96 for a confidence level of 95%.

$$n=\frac{\text{N}\text{*}\text{X}}{(\text{X}+\text{N}-1)} where X=\frac{1.96\text{*}\text{p}\text{*}\left(1-\text{p}\right)}{{\left(\text{m}\text{a}\text{r}\text{g}\text{i}\text{n} \text{o}\text{f} \text{e}\text{r}\text{r}\text{o}\text{r}\right)}^{2}}$$

It was determined that a minimum of 347 respondents per country was required. It was therefore decided to target 375 respondents per country, as a conservative measure. The survey was closed to further participants once this target number had been reached.

### Analysis of survey results

Data collection, cleaning and analysis was performed by three members of the SwipeRx research and public health team. Results were exported from Qualtrics into Microsoft Excel, and any necessary case duplication removal (verified by the unique SwipeRx user identifier) was performed. Responses were categorized as urban or rural depending on the district and province provided by each respondent. Only responses that were ≥ 80% complete were included in analyses. Data were then analysed using STATA 16 software (StataCorp, College Station, TX, USA). Data are presented as medians for continuous responses and percentages/percentage distribution for categorical responses. Potential differences between responses from urban and rural respondents were assessed using Chi-Square tests for categorical data and Mann-Whitney U tests for numerical data.

### Development, implementation and evaluation of the CPD module

The gap in pharmacy professionals’ knowledge identified by the survey highlighted a need for additional educational materials on diabetes for pharmacists in Cambodia and Viet Nam. A digital CPD module was therefore developed. The content of the module was informed by an overarching CPD outline and narrative content, both developed in English (Supplementary File 2). The narrative was consistent with technical guidance from FIND and local Ministries of Health, and global best practices relevant to digital education and pharmacy professional behaviour change. Using the final English narrative, country-specific content, including pre/post knowledge assessment questions and a number of interactive exercises throughout the module, was then tailored and translated into local language and format consistent with guidance from local accreditation partners, namely the Pharmacy Council of Cambodia, and the Ho Chi Minh City Pharmacy Association. In Cambodia, the final module was deployed using an illustrated narrative format, and in Viet Nam, a webinar format was utilized in line with local regulations.

The objectives of the module were to enable users to: identify risk factors for as well as signs and symptoms of diabetes; counsel clients regarding 4 M (monitoring, medication, meals and movement) recommended diabetes management strategies; understand why accurate BGM products are important, and what factors can affect the accuracy of BGM systems; use BGM meter and strip products safely and appropriately; help clients interpret glucose levels and identify cases where further care is needed at a health facility; counsel clients about co-morbidity risks for diabetes, particularly COVID-19; understand and explain key diabetes concepts and terms often used by healthcare professionals.

The module was made available to pharmacy professionals and pharmacy students on SwipeRx, and promoted through SwipeRx social media channels and by pharmacist key opinion leaders in both countries. The estimated completion time was 60–120 min. Users answered knowledge assessment questions (n = 14 in Cambodia and n = 10 in Viet Nam) before and after completing the module. Users were required to correctly answer ≥ 60% (Cambodia) or ≥ 70% (Viet Nam) of post-CPD knowledge assessment questions to pass the assessment, consistent with local accreditation partner requirements. In accordance with accreditation guidelines, only pharmacists and pharmacy assistants could receive accredited CPD points for passing the assessment.

### Analysis of CPD module

Data are presented as percentages of users who correctly answered each question.

## Results

### Respondent characteristics

In total, 386 pharmacy professionals from Cambodia and 375 from Viet Nam completed the digital survey (Table 1). Most of the respondents came from urban districts (≥ 71%), and the majority were female (≥ 76%), were pharmacists (≥ 80%), and worked at independent retail pharmacies (≥ 86%).

**Table 1:** Respondent characteristics

	Cambodia (N = 386)	Viet Nam (N = 375)
Provinces covered, n/N	20/25	25/63
Female, %^a^	79	76
Age (years), median (interquartile range)	28 (26–32)	31 (26–36)
Setting, %		
Urban	71	74
Rural	29	26
Profession, %^b^		
Pharmacist	81	80
Pharmacy assistant	8	3
Pharmacy owner	17	21
Pharmacy manager	49	25
Workplace type, %^c^		
Independent retail pharmacy	90	86
Chain retail pharmacy	5	6
Pharmacy at public health facility	1	1
Pharmacy at private health facility	4	7

^a^N=385 for Cambodia; ^b^Respondents could report multiple roles; ^c^N=374 for Viet Nam

In Cambodia, 69% of respondents reported stocking blood glucose meters and test strips and 91% reported stocking oral antidiabetic medication (OAM). Less than 1 in 5 (18%) reported stocking insulin and only 14% reported stocking all four types of diabetes products. In Viet Nam, 88% of respondents reported stocking blood glucose test strips, 65% reported stocking blood glucose meters, 82% reported stocking OAM, and 40% reported stocking insulin; 30% of respondents reported stocking all four types of diabetes products. In Cambodia and Viet Nam, more respondents in urban pharmacies reported stocking blood glucose meters or insulin compared with rural respondents, and in Viet Nam only, more respondents in urban pharmacies reported stocking blood glucose test strips (Supplementary File 3).

### Volume of diabetes-related clients

Overall, 71% of respondents from Cambodia and 50% from Viet Nam reported an average number of clients for diabetes-related products of 1 to 5 clients per week, 20% and 24% reported 6–10 clients per week, 4% and 11% reported 11–20 clients per week, and 5% and 14% reported > 20 clients per week.

In Cambodia, respondents reported that a median of 3 clients per week purchase blood glucose test strips and insulin, and 1 client purchases blood glucose meters. Corresponding values for Viet Nam were 8 clients for blood glucose test strips, 7 clients for insulin and 3 clients for blood glucose meters. In Cambodia and Viet Nam, respondents from urban settings reported higher volumes of blood glucose meter and insulin clients than those from rural settings (Supplementary File 3). In Cambodia, 3% reported client purchase of blood glucose test strip purchase once a week, 10% 2–3 times a month, 35% once a month and 44% once every few months. Corresponding values for insulin purchase were 6%, 20%, 39% and 25%. In Viet Nam, 2% reported client purchase of blood glucose test strip purchase once a week, 13% 2–3 times a month, 41% once a month and 42% once every few months. Corresponding values for insulin purchase were 8%, 18%, 50% and 21%.

### Diabetes knowledge, attitudes and practices

Overall, 33% of respondents from Cambodia and 63% from Viet Nam reported that they conduct in-pharmacy glucose monitoring. In total, 84% of respondents from Cambodia and 62% from Viet Nam reported that they provided counselling to diabetes clients, and 70% and 54%, respectively, reported that they referred clients to healthcare facilities for diabetes care.

**Fig. 1:**
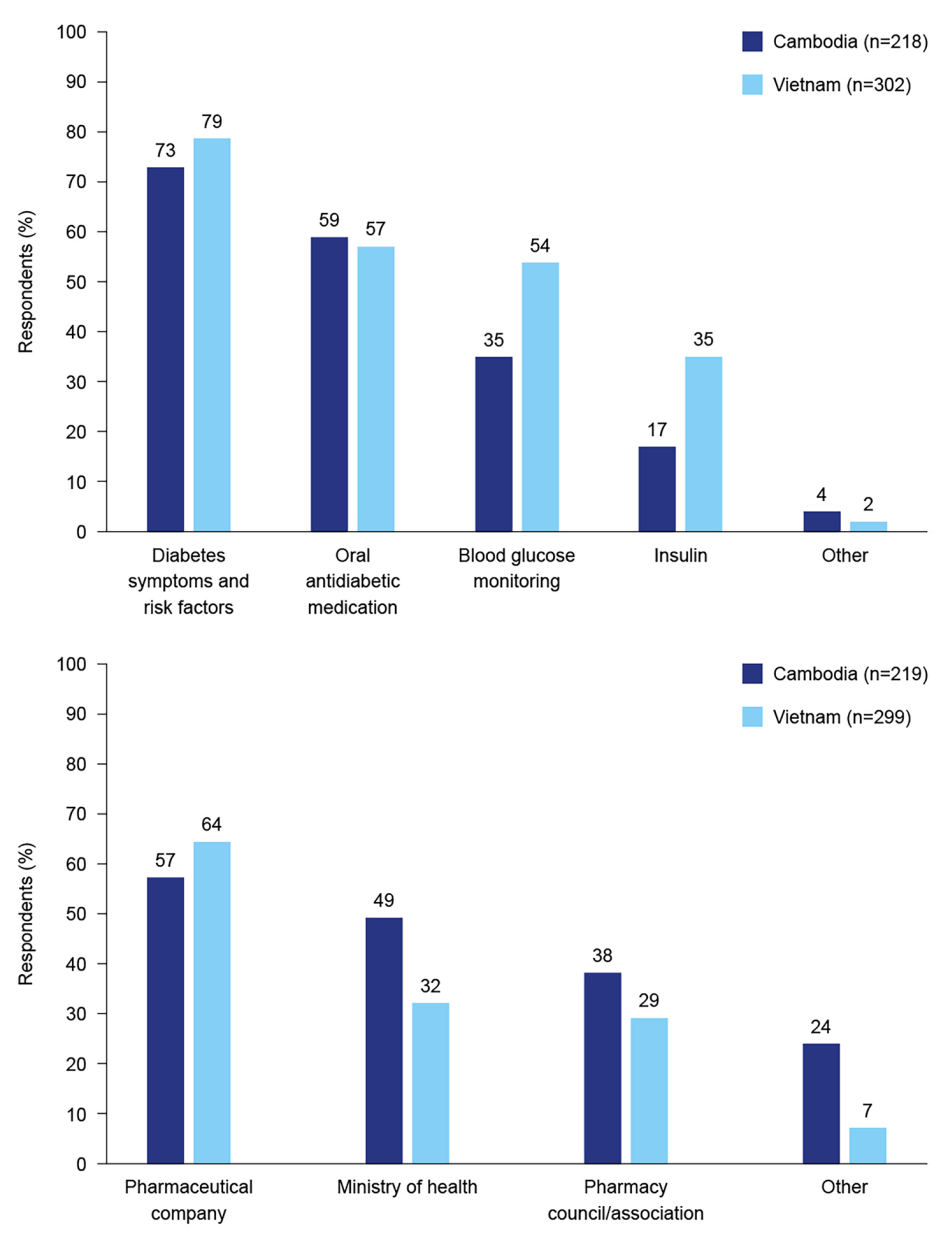
Topics and sources of diabetes education among respondents who reported receiving education during the past three years

In total, 57% of respondents from Cambodia and 81% of respondents from Viet Nam reported receiving some education about diabetes in the past three years. Sources of diabetes professional education in Cambodia and Viet Nam included pharmaceutical companies, Ministries of Health and pharmacy associations (Fig. 1). For those who had received education, topics included diabetes symptoms and risk factors, OAM, blood glucose monitoring and insulin (Fig. 1).

When asked the question “for a client diagnosed with diabetes and prescribed multiple daily doses of insulin, how frequently should they check their blood glucose level?“ with possible answers being once a month, once a week, once a day, several times per day, and don’t know, only 19% in Cambodia and 14% in Viet Nam indicated the correct answer as being several times per day (Supplementary File 4).

In Cambodia, urban respondents were more likely to have been educated about OAM or BGM than rural respondents and were more likely to have participated in education sponsored by a pharmaceutical company (Supplementary File 3). In Viet Nam, respondents from rural districts were more likely to have been educated about diabetes symptoms and risk factors, while urban respondents were more likely to have been educated about OAM or BGM. Rural respondents from Viet Nam were more likely to have received diabetes education from pharmaceutical companies compared with urban respondents.

**Fig. 2:**
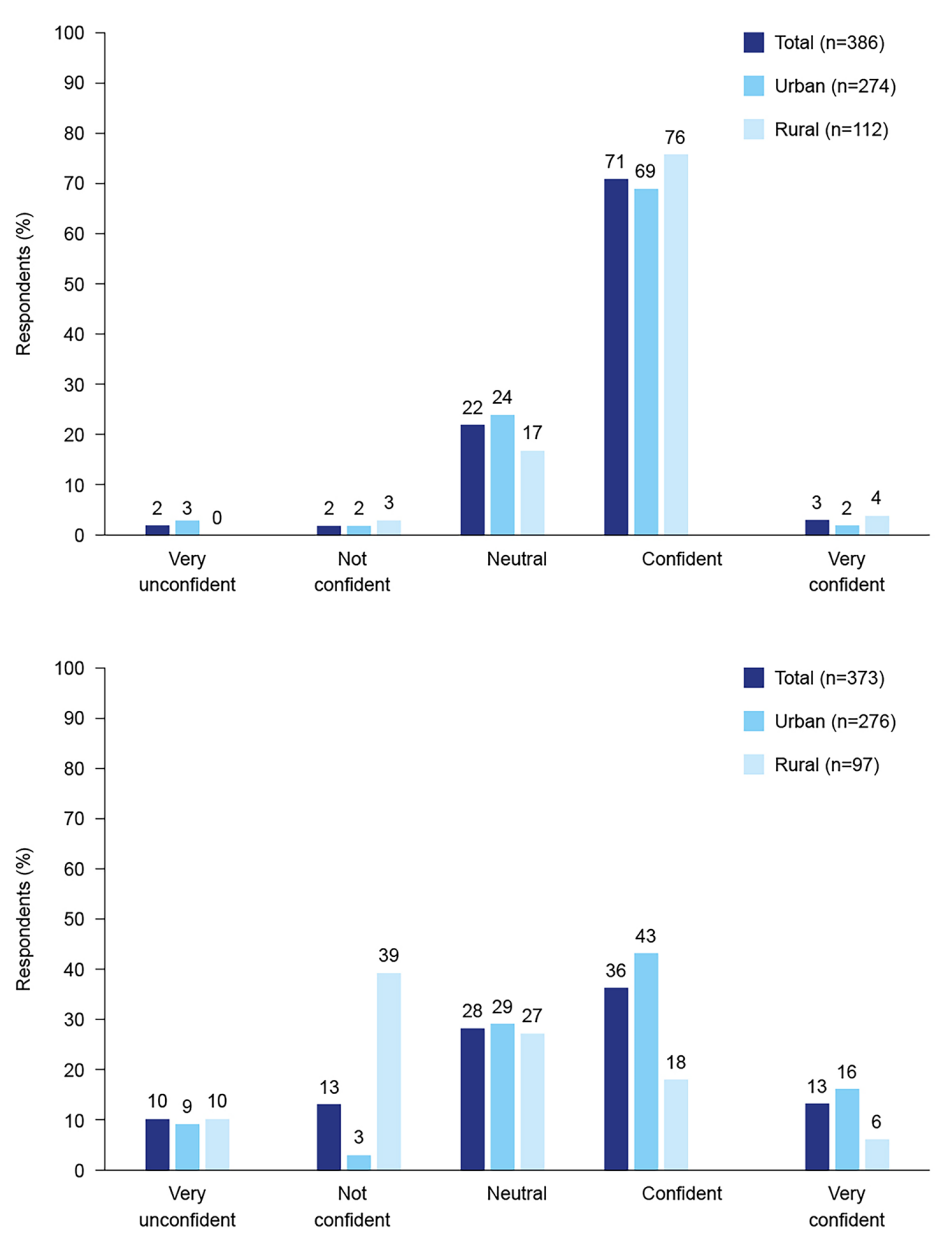
Respondents’ level of confidence in ability to provide blood glucose monitoring product advice to clients in (A) Cambodia (upper panel) and (B) Viet Nam (lower panel)

**Fig. 3:**
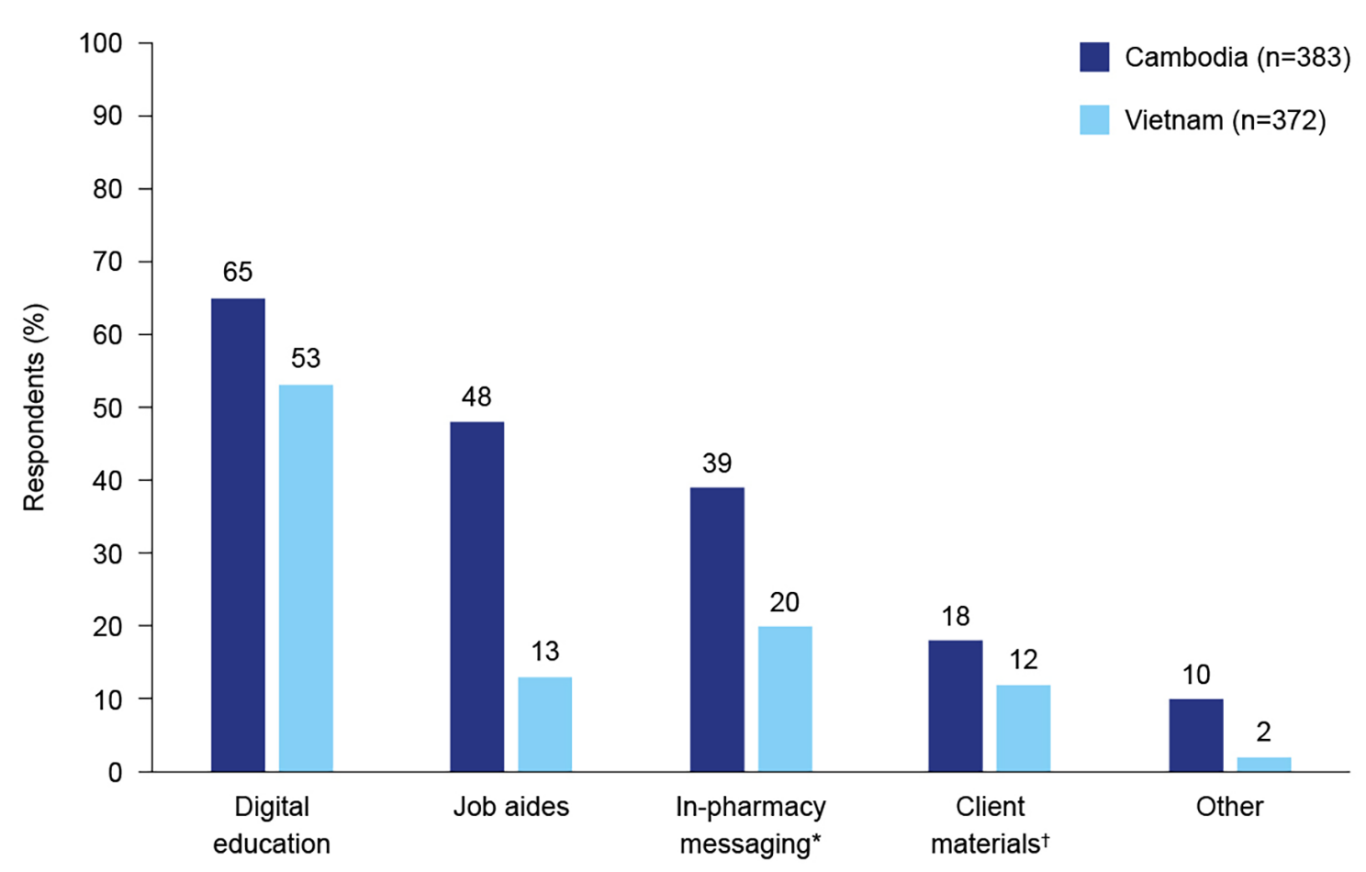
Respondents’ preferred methods of improving confidence in counselling clients about blood glucose monitoring. E.g. poster, dangler; †e.g. QR code to website with information

Most respondents (74%) in Cambodia described themselves as confident or very confident in providing BGM advice and information to their clients, compared with only 49% of respondents from Viet Nam (Fig. 2). In Viet Nam, pharmacists in rural areas were less confident in providing BGM advice to their clients, compared with those in urban areas. Preferred methods to improve confidence in counselling diabetes clients about BGM were digital education, job aides and in-pharmacy messaging (Fig. 3). Questions commonly asked by clients at the pharmacy fell into four categories: safety and side effects of diabetes medications, diabetes symptoms and causes, blood glucose level targets and acceptable levels, and diet and exercise (Supplementary File 5). Questions included “how do I know whether I have diabetes?”, “what are the symptoms of diabetes?”, “what factor/s cause diabetes?“, “do I need to take diabetes medications for life, and if so, what are the health risks associated with long-term use?“, “do you have guidance for injecting or taking oral diabetes drugs?“, and “will my blood glucose level decrease after using the medication?”.

Factors considered by the survey respondents when recommending BGM products to clients are shown in **Supplementary File 6**. Product quality of BGM test strips and meters was considered by the highest proportion of respondents in both countries; brand recognition and price were also frequently considered.

### CPD module knowledge assessment

In Cambodia, the module was made accessible for a 9-week period (26 November 2021 to 31 January 2022). During this time, 1,276 pharmacy professionals/students accessed the module. In total, 1,137 (89%) attempted the post-CPD knowledge assessment questions, of whom 1,124 (99%) passed. Of these 1,124 individuals, 832 (74%) were pharmacists and 911 (81%) were female. In total, 859 pharmacy professionals received accredited CPD points.

In Viet Nam, feedback from users regarding format and length led to revision and relaunch of the module as a shortened webinar. The number of knowledge assessment questions was reduced from 14 to 10. Additionally, due to limited uptake during the Lunar New Year period, access to the module was extended, with a total access period of 15 weeks (17 December 2021 to 31 March 2022). In total, 524 pharmacy professionals/students accessed the module, of whom 399 (76%) attempted the post-CPD knowledge assessment questions. Of these, 376 passed the assessment; 316 (84%) were pharmacists and 264 (70%) were female. In total, 188 pharmacy professionals/students received accredited CPD points.

**Fig. 4:**
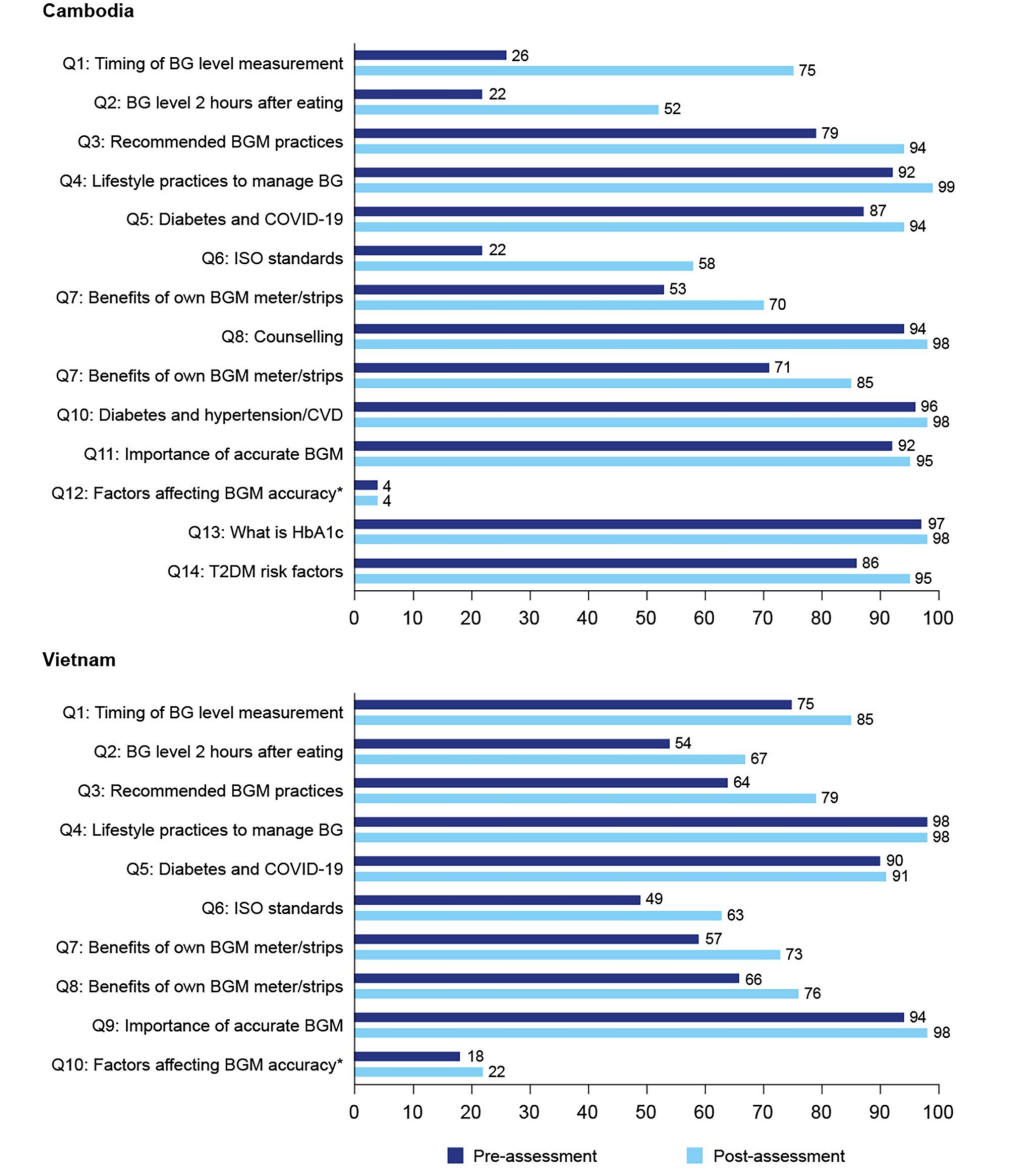
Percentage of users providing correct answers to the CPD knowledge assessment questions pre- and post-module in (A) Cambodia and (B) Viet Nam. BG, blood glucose; COVID-19, coronavirus disease 2019; CVD, cardiovascular disease; HbA1c, glycated haemoglobin; ISO, International Organization for Standardization; T2DM, type 2 diabetes mellitus.*Question was “Which factors are not relevant to the accuracy of BGM results?”. The low percentage of pre- and post-assessment correct answers suggest that users may have misread this as “which factors ARE relevant to the accuracy of BGM results?”. In addition, it is likely that the module content was not sufficiently clear about factors affecting BGM accuracy

Knowledge levels post-CPD improved, with percentages of individuals providing correct answers increasing substantially compared with pre-CPD levels for 10 of 14 questions in Cambodia and 6 of 10 questions in Viet Nam (Fig. 4). These included questions on timing of measurement of blood glucose levels, recommended BGM practices, International Organization for Standardization (ISO) standards, benefits of owning BGM products, and advice on expired BGM strips.

For the remaining questions, the percentage of individuals answering correctly was high pre-CPD, leaving limited room for improvement. An exception to this was the question about factors that are not relevant to the accuracy of BGM results, which was answered correctly by only 4% pre- and post-CPD in Cambodia, and 18% pre- and 22% post-CPD in Viet Nam.

## Discussion

Most respondents to this 2021 survey of pharmacy professionals in Cambodia and Viet Nam reported stocking BGM supplies and OAM. However, although more than half of the respondents described themselves as confident in providing BGM advice to clients, and the majority reported receiving some education about diabetes within the past three years, the extent and quality of this education appeared to be limited based on reported knowledge and practices. An accredited, digital CPD module developed to address knowledge gaps was able to reach a large number of pharmacy professionals and students in a short time period, and resulted in substantial improvements in users’ knowledge of diabetes management and BGM.

The survey results identified gaps in pharmacy professionals’ knowledge of and practices related to diabetes. While a substantial proportion of pharmacy professionals reported having received some diabetes education in the last three years, knowledge on the recommended frequency of BGM was limited. This may be due to insufficient focus on BGM in previous trainings: only one-third to one-half of recently educated respondents reported that BGM was included in their training/education. Additionally, as most of the education received prior to the SwipeRx CPD was provided by pharmaceutical companies, it may have been focussed on specific products or therapies, rather than general disease awareness and monitoring. This data highlights a need for comprehensive capacity building including multiple CPDs and post-CPD follow-up to promote adherence with quality standards for pharmacy professionals in Southeast Asia to ensure that they are well positioned to recognize potential symptoms of disease and to provide comprehensive and accurate information on self-management practices, particularly as people living with diabetes often approach their pharmacy for information on a range of topics related to diabetes, as shown by our survey. Consistent with our findings, previous studies in other countries have highlighted gaps in pharmacists’ diabetes-related knowledge, highlighting the importance of continuing education [11, 12].

Although respondents from urban pharmacies in our survey were more likely to stock blood glucose meters and insulin, reported higher volumes of blood glucose meter and insulin clients, and were more likely to have been educated about OAM or BGM compared with rural respondents, the majority of rural respondents from Cambodia were confident in providing BGM advice. In Viet Nam, however, more than 50% of rural respondents described themselves as neutral or not confident in providing BGM advice. As the difference in prevalence of diabetes between urban and rural areas in Asian and Pacific countries may be narrowing [2], the need for diabetes education for pharmacists in rural areas may become increasingly important.

More than half of the survey respondents reported digital education as their preferred method of improving confidence in providing BGM counselling to clients. This is consistent with the high uptake levels of the CPD module. This high level of engagement demonstrates that SwipeRx-user pharmacists in Cambodia and Viet Nam are highly motivated and willing to improve their knowledge of diabetes and BGM practices. The fact that the CPD module resulted in substantial improvements in diabetes knowledge and was able to educate many pharmacy professionals and students in a short time shows that digital education platforms can rapidly upskill and empower pharmacy professionals at scale. Our findings are consistent with a previous study that demonstrated the benefits of a targeted online diabetes training tool for pharmacists in Malaysia and Australia [13]. The low numbers of pharmacy professionals who correctly answered the question about factors that are not relevant to the accuracy of BGM results (4% in Cambodia and 22% in Viet Nam) may have been due to users misreading the question and selecting factors that are relevant to the accuracy of BGM results, or because module content was not sufficiently clear about factors affecting BGM accuracy. This highlights the importance of clarity and ease of understanding in educational materials.

Pharmacists are one of the most trusted professions worldwide, alongside nurses, teachers, firefighters and doctors [5, 14], as they are located within the community, are accessible, and have an informal set up without paperwork or other administrative requirements [5, 6]. As a result, they have potential to provide healthcare support for people when they need it the most, especially for conditions such as diabetes that require continuous self-management [8, 15–17]. Every interaction with pharmacy clients represents an opportunity for community pharmacists to empower people living with diabetes to better manage their condition [5]. The breadth of questions from clients reported by our survey respondents demonstrates that pharmacists are seen as an important source of advice and guidance for communities. However, our survey showed that the reported volume of diabetes clients in pharmacies in Cambodia and Viet Nam is relatively low when considering the overall prevalence of diabetes in these countries, suggesting that the potential of pharmacists as key healthcare providers for patients with diabetes is not being optimally realised. This may be due to a misconception that their role is restricted to that of a dispensing agent, as well as limited community awareness about the need for regular BGM and the potential to receive diabetes management support from trained community pharmacists. There is an opportunity for pharmacists in these countries to establish themselves as key providers of quality diabetes supplies, education and support, and potentially to provide additional services such as screening programmes and disease awareness campaigns.

The reasons behind the poor accessibility of insulin in our survey were not explored. Potential reasons include cost (for clients), limitations in the pharmacy supply system, lack of clinician training leading to low prescribing practices, and/or client preference [18]. It could also be an indication of clinical inertia, which has been described as an enemy of therapeutic success in the management of diabetes and its complications [19]. In Viet Nam, antidiabetic medication is available at low cost or free of charge with health insurance, thus these products are less likely to be stocked in private pharmacies [20]. Additionally, injectable medications may be more likely to be received in hospital settings than from pharmacies. Regardless, our findings highlight a potential need for improved access to insulin at pharmacies in Cambodia and Viet Nam.

According to our survey, pharmacy professionals’ recommendations for BGM products are based mainly on quality, brand and price. In Cambodia, recommendations from clinicians and product promotions also influenced pharmacy professional recommendations. Understanding how quality is evaluated was not part of this survey; further research to determine whether pharmacy professionals require support to make appropriate recommendations for BGM products would be of value.

There are several limitations to this study. A high proportion of respondents were employed at independent retail pharmacies, thus the results may not be fully representative of the experiences of pharmacy professionals working at chain retail pharmacies or health facilities. Furthermore, only users of the SwipeRx platform could participate. There was no limit on number of responders per pharmacy and information needed to determine whether multiple respondents from the same pharmacy completed the survey was not collected through the survey. Additionally, the findings may not be applicable to other countries in Southeast Asia due to differences in professional education regulations and SwipeRx coverage. Participants may have been pre-disposed to self-selection bias due to their interest on diabetes and blood glucose monitoring, and thus may have been more knowledgeable of the topics presented in the survey compared to other SwipeRx-users. The pre- and post-CPD knowledge assessment questions represent self-reported levels of knowledge, and no validation testing was performed. A follow-on survey would be required to assess longer-term changes in knowledge. Finally, it is acknowledged that learning and upskilling is not a once-off process; nevertheless, this initial CPD module had a meaningful impact in terms of both knowledge improvement and reach. Other strengths of the study include the survey’s large sample size and high response rate, and inclusion of viewpoints from both urban and rural provinces.

## Conclusions

This study identified gaps in pharmacists’ knowledge of and practices related to diabetes in Cambodia and Viet Nam. An accredited digital CPD module developed to address knowledge gaps resulted in improvements to users’ knowledge of diabetes management and BGM. Pharmacists are a vital link in community-based programmes for diabetes care in Southeast Asia. Continuing to invest in digital education and post-education capacity building for pharmacy professionals to address knowledge gaps could strengthen access to comprehensive and accurate information on diabetes management at scale, and quality BGM products and services, for people living with diabetes in Southeast Asia.

## Electronic supplementary material

Below is the link to the electronic supplementary material.

Supplementary Material 1

## Data Availability

The dataset(s) supporting the conclusions of this article is(are) available in the Zenodo repository (DOI 10.5281/zenodo.7347940, https://zenodo.org/record/7347940#.Y33tpXbP1PZ).

## Abbreviations

BG: blood glucose
BGM: blood glucose monitoring
CPD: continuing professional development
CVD: cardiovascular disease
COVID-19: coronavirus disease 2019
HbA1c: glycated haemoglobin
ISO: International Organization for Standardization
OAM: oral antidiabetic medication
T2DM: type 2 diabetes mellitus
