# Supplementary material for: Diabetes and blood glucose monitoring knowledge and practices among pharmacy professionals in Cambodia and Viet Nam: digital survey and education
Source: BMC Med Educ. 2023 Jun 29;23:483. doi: 10.1186/s12909-023-04449-0 (PMC10308732; doi:10.1186/s12909-023-04449-0)

# Supplementary Files

**Supplementary File 1.** The online survey

## Screening questions

| Var Name | Question and Responses | Code and Logic |
| --- | --- | --- |
| S1 | Do you participate in any of the following activities at your pharmacy? (select all that apply) | END SURVEY if either: (1) S1=a or b is NOT selected, or  (2) S1=e is selected |
|  | 1. Ordering and purchasing products and supplies to be sold at the pharmacy | 1 |
|  | 1. Dispensing medications to clients | 2 |
|  | 1. Counselling and providing clients advice on products | 3 |
|  | 1. Other services in the pharmacy | 4 |
|  | 1. I don’t work in a pharmacy | 5  If S1=d is selected, end survey |
| S2. | Do you stock BGM or other diabetes-related products at the pharmacy? | END SURVEY if S2=1 (“No”) |
|  | 1. No | 0 |
|  | 1. Yes | 1 |

## Survey questions

| Var Name | Question and Responses | Code and Logic |
| --- | --- | --- |
| DEMOGRAPHICS | | |
| D1 | What province do you work in? | (drop down: all provinces) |
| D2 | Which district do you work in? | Select from list |
| D3 | What is your gender? |  |
|  | 1. Female | 1 |
|  | 1. Male | 2 |
|  | 1. Other/I’d prefer not to say | 3 |
| D4 | How old are you? | Scale: 1-100  If age selected<18, end survey |
| D5 | What is your current professional role/s in the pharmacy? (select all that apply) |  |
|  | 1. Pharmacist | 1 |
|  | 1. Pharmacy assistant | 2 |
|  | 1. Pharmacy manager | 3 |
|  | 1. Pharmacy owner |  |
|  | 1. Other, please specify: [open text] | 4  If D5=4 is selected and D5=1-3 is NOT selected, end survey |
| D6 | What type of pharmacy is your main workplace type? |  |
|  | 1. Independent retail pharmacy | 1 |
|  | 1. Chain retail pharmacy | 2 |
|  | 1. Online pharmacy | 3 |
|  | 1. Pharmacy at a public health facility | 4 |
|  | 1. Pharmacy at a private health facility | 5 |
| CLIENT VOLUME | | |
| CV1 | How many pharmacy clients does your pharmacy receive on average per *day*? |  |
|  | 1. 1-20 clients per day | 1 |
|  | 1. 21-50 clients per day | 2 |
|  | 1. 51-100 clients per day | 3 |
|  | 1. More than 100 client per day | 4 |
| CV2 | On average, how many pharmacy clients come for diabetes-related products (BGM or medication) per *week*? |  |
|  | 1. 1-5 clients per week | 1 |
|  | 1. 6-10 clients per week | 2 |
|  | 1. 11-20 clients per week | 3 |
|  | 1. More than 20 clients per week | 4 |
| CV3 | Among all of your clients seeking diabetes products (any type), approximately how many of those clients purchase the following blood glucose monitoring products *per week*? |  |
| CV3_1 | Blood glucose test strips | Number scale: 0-200 (# weekly clients); force response and add ‘refuse to answer option’ |
| CV3_2 | Blood glucose test meters | Number scale: 0-200 (# weekly clients); force response and add ‘refuse to answer option’ |
| CV4 | Among all of your clients seeking diabetes products (any type), approximately how many of those clients purchase insulin products *per week*? | Number scale: 0-200 (# weekly clients);  force response and add ‘refuse to answer option’ |
| CV5 | How do most clients purchase blood glucose test strips at your pharmacy? |  |
|  | 1. Most clients purchase individual or packs of 10 test strips | 1 |
|  | 1. Most clients purchase larger packs of 25 or 50 test strips less | 2 |
|  | 1. Most clients purchase packs of 100 test strips or more | 3 |
| CV6 | In general, how frequently do clients purchase blood glucose test strips? |  |
|  | 1. Once every few months | 1 |
|  | 1. Once a month | 2 |
|  | 1. 2-3 times a month | 3 |
|  | 1. Once a week | 4 |
|  | 1. Other | 5 |
| CV7 | In general, how frequently do clients purchase insulin? |  |
|  | 1. Once every few months | 1 |
|  | 1. Once a month | 2 |
|  | 1. 2-3 times a month | 3 |
|  | 1. Once a week | 4 |
|  | 1. Other | 5 |
| Diabetes Care and BGM Product Knowledge | | |
| K1 | For a client diagnosed with diabetes prescribed multiple daily doses of insulin, how frequently should they check their blood glucose level? |  |
|  | 1. Once a month | 1 |
|  | 1. Once a week | 2 |
|  | 1. Once a day | 3 |
|  | 1. Several times in the day | 4 |
|  | 1. Don’t know | 9  Make exclusive option |
| K2 | Have you received training or education on diabetes-related care or products within the last 3 years? If so, please indicate the topics. (select all that apply) |  |
|  | 1. Diabetes symptoms and risk factors | 1 |
|  | 1. Insulin | 2 |
|  | 1. Anti-diabetes medication | 3 |
|  | 1. Blood glucose monitoring products | 4 |
|  | 1. Other, please specify: [open text] | 5 |
|  | 1. I have not received diabetes-related training or education in the past 3 years | 6  Make exclusive option |
| K3 | Who provided the diabetes-related education? (select all that apply) |  |
|  | 1. MOH | 1 |
|  | 1. Pharmacy Council/Association | 2 |
|  | 1. Pharmaceutical company | 3 |
|  | 1. Other, please specify: [open text] | 4 |
| Diabetes Care and BGM Product Attitudes | | |
| A1 | Which factors most influence your decision to recommend one blood glucose monitoring brand over another? (select a maximum of 3) |  |
|  | 1. Brand reputation | 1 |
|  | 1. Price | 2 |
|  | 1. Margin | 3 |
|  | 1. Client’s request | 4 |
|  | 1. Other, please specify: [open text] | 5 |
| A2 | How confident are you in your ability to provide informed advice on blood glucose monitoring products to clients? |  |
|  | 1. Very not confident | 1 |
|  | 1. Not confident | 2 |
|  | 1. Neutral | 3 |
|  | 1. Confident | 4 |
|  | 1. Very confident | 5 |
| A3 | What can make you more confident to provide informed advice on blood glucose monitoring products to clients? (Please be specific) |  |
|  | 1. Digital education | 1 |
|  | 1. Job aides | 2 |
|  | 1. In-pharmacy messaging (i.e., poster, dangler) | 3 |
|  | 1. Client materials (i.e., QR code to website with information) | 4 |
|  | 1. Other, please specify: [open text] | 5 |
| Diabetes Practices and Supply Chain | | |
| P1 | Which of the following activities related to diabetes do you currently participate in? (select all that apply) |  |
|  | 1. In-pharmacy glucose monitoring for diabetes | 1 |
|  | 1. Referring clients for diabetes care at a health facility | 2 |
|  | 1. Counselling diabetes clients | 3 |
|  | 1. Other, please specify: [open text] | 6 |
|  | 1. None of the above | 7  Make exclusive option |
| P2 | What are some questions that clients ask you related to diabetes and diabetes products? | Open text |
| P3 | Which of the following do you sell in your pharmacy? (Select all that apply) |  |
|  | 1. Blood glucose test strips | 1 |
|  | 1. Blood glucose -meters | 2 |
|  | 1. Insulin | 3 |
|  | 1. Oral anti-diabetic medication | 4 |
| P4 | Can you list your top 3 selling blood glucose monitoring (BGM) TEST STRIP brands that you sell in your pharmacy and the package size of the product (i.e. Lifescan, pack of 10 strips)? | Will appear if P3=a was selected |
|  | 1. Number 1 (best) BGM TEST STRIP top seller and package size: [open text]] | 1 |
|  | 1. Number 2 BGM TEST STRIP top seller and package size: [open text] | 2 |
|  | 1. Number 3 BGM TEST STRIP top seller and package size: [open text] | 3 |
| P5 | On average, how many units of your top 3 best-selling BGM TEST STRIPS products do you sell per week? | Will appear if P3=a was selected |
|  | 1. Weekly sales of number 1 (best) BGM strip top seller | Number scale |
|  | 1. Weekly sales of number 2 BGM strip top seller | Number scale |
|  | 1. Weekly sales of number 3 BGM strip top seller | Number scale |
| P6 | What are your purchase prices of your top 3 best-selling BGM TEST STIP products? (buy-in price from supplier) | Will appear if P3=a was selected |
|  | 1. Price ($) of number 1 (best) BGM strip top seller | Number scale ($) |
|  | 1. Price ($) of number 2 BGM strip top seller | Number scale ($) |
|  | 1. Price ($) of number 3 BGM strip top seller | Number scale ($) |
| P7 | What are the selling prices of your top 3 best-selling BGM -TEST STRIP products? | Will appear if P3=a was selected |
|  | 1. Price ($) of number 1 (best) BGM strip top seller | Number scale ($) |
|  | 1. Price ($) of number 2 BGM strip top seller | Number scale ($) |
|  | 1. Price ($) of number 3 BGM strip top seller | Number scale ($) |
| P8 | What are the most influential factor/s influencing sales of BGM TEST STRIP products? (select max 3) | Will appear if P3=a was selected |
|  | 1. Brand recognition | 1 |
|  | 1. Product quality | 2 |
|  | 1. Price | 3 |
|  | 1. Promotions on products | 4 |
|  | 1. Recommendation of products from clinicians | 5 |
|  | 1. Other, please specify: [open text] | 6 |
| P9 | What are some other items that clients frequently purchase along with blood glucose test strips? (select all that apply) | Open text; Will appear if P3=a was selected |
|  | 1. Insulin | 1 |
|  | 1. Oral anti-diabetic medication | 2 |
|  | 1. Lancets or meters | 3 |
|  | 1. Other, please specify: [open text] | 4 |
|  | 1. None of the above | 5  Make exclusive option |
| P10 | Can you list your top 3 selling blood glucose monitoring (BGM) meter brands that you sell in your pharmacy? | Will appear if P3=b was selected |
|  | 1. Number 1 (best) BGM meter top seller: [open text] | 1 |
|  | 1. Number 2 BGM meter top seller: [open text] | 2 |
|  | 1. Number 3 BGM meter top seller: [open text] | 3 |
| P11 | On average, how many units of your top 3 best-selling BGM meters do you sell per week? | Will appear if P3=b was selected |
|  | 1. Weekly sales of number 1 (best) BGM meter top seller | Number scale |
|  | 1. Weekly sales of number 2 BGM meter top seller | Number scale |
|  | 1. Weekly sales of number 3 BGM meter top seller | Number scale |
| P12 | What are your purchase prices of your top 3 best-selling BGM meters? (buy-in price from supplier) | Will appear if P3=b was selected |
|  | 1. Price ($) of number 1 (best) BGM meter top seller | Number scale ($) |
|  | 1. Price ($) of number 2 BGM meter top seller | Number scale ($) |
|  | 1. Price ($) of number 3 BGM meter top seller | Number scale ($) |
| P13 | What are the selling prices of your top 3 best-selling BGM meters? | Will appear if P3=b was selected |
|  | 1. Price ($) of number 1 (best) BGM meter top seller | Number scale ($) |
|  | 1. Price ($) of number 2 BGM meter top seller | Number scale ($) |
|  | 1. Price ($) of number 3 BGM meter top seller | Number scale ($) |
| P14 | What are the main factors influencing sales of BGM meter products? (select max 2) | Will appear if P3=b was selected |
|  | 1. Brand recognition | 1 |
|  | 1. Product quality | 2 |
|  | 1. Price | 3 |
|  | 1. Promotions on products | 4 |
|  | 1. Recommendation of products from clinicians | 5 |
|  | 1. Other, please specify: [open text] | 6 |
| SCP1 | How many suppliers (i.e. wholesalers or distributors) do you purchase blood glucose meters and test strips - from? | Number scale (1-20) |
| SCP2 | For blood glucose meters and test strips-, on average, how often do you make purchases from these suppliers (i.e. wholesalers or distributors)? |  |
|  | 1. Less than once a month | 1 |
|  | 1. Between 1-3 times a month | 2 |
|  | 1. Once a week | 3 |
|  | 1. Several times a week | 4 |
|  | 1. Everyday | 5 |
| SCP3 | Who do you purchase blood glucose monitoring products from? | Open text or name & distributor/wholesaler if known; Will appear if P3=a was selected |
| SCP4 | Are the suppliers (i.e. wholesalers or distributors) you purchase glucose monitoring products (BGM) and insulin products from the same or different? | Will appear if P3=a AND P3=b or c was selected |
|  | 1. No, none of the BGM and insulin products are from the same suppliers | 1 |
|  | 1. Some are from the same suppliers | 2 |
|  | 1. Most are from the same suppliers | 3 |
|  | 1. Yes, all BGM and insulin products are from the same suppliers | 4 |
| SCP5 | How do you buy blood glucose monitoring products from suppliers? (select all that apply) | Will appear if P3=a was selected |
|  | 1. Purchase from salespeople who visit my pharmacy | 1 |
|  | 1. Purchase online | 2 |
|  | 1. Purchase by phone | 3 |
|  | 1. Physically visit wholesaler or distributor to purchase | 4 |
|  | 1. Other, please specify: [open text] | 5 |
| SCP6 | How many times this year (since January 2021) have you had blood glucose monitoring products on the shelf expire? |  |
|  | - - - 1. Never | 1 |
|  | - - - 1. Once | 2 |
|  | 1. More than once | 3 |
| SCP7 | How often in the past month did you experience stock out of blood glucose monitoring products at your pharmacy? | Number scale (0-50); Will appear if P3=a was selected |
| SCP8 | What type of incentives have been provided by suppliers for any products at your pharmacy? (select all that apply) |  |
|  | 1. Discounts | 1 |
|  | 1. Credit | 2 |
|  | 1. Free product promos (i.e. get the first couple free) | 3 |
|  | 1. Pharmacy equipment (i.e. scales, medicine cabinets, etc.) | 4 |
|  | 1. Other, please specify: [open text] | 5 |
|  | 1. No incentives have been provided | 6 |
| SCP9 | What type of incentives have been provided by suppliers specifically for blood glucose monitoring products? (select all that apply) | Will appear if P3=a was selected |
|  | 1. Discounts | 1 |
|  | 1. Credit | 2 |
|  | 1. Free products promos (i.e. get the first couple free) | 3 |
|  | 1. Pharmacy equipment (i.e. scales, medicine cabinets, etc.) | 4 |
|  | 1. Other, please specify: [open text] | 5 |
|  | 1. No incentives have been provided | 6  Make exclusive option |
| Feedback on New BGM Market Models | | |
| F1 | Would you be interested in participating in a future program to improve access to quality, affordable blood glucose monitoring products? |  |
|  | - 1. Yes | 1 |
|  | - 1. No | 2 |
| F2 | If you answered “yes”, do you consent for SwipeRx to contact you? If so, please provide the best ways to contact you |  |
|  | 1. No, I don’t consent | 1 |
|  | 1. Yes, I consent. My contact info is: [open text] | 2 |

**Supplementary File 2.** Continuing professional development module narrative content

|  | **Module 1: Diabetes Overview** | **Reference Materials** |
| --- | --- | --- |
| **Section 1: Diabetes 101** | | |
| - 1. Intro | In Cambodia, 430,600 people or 4.4% of the adult population are living with diabetes, a percentage that is increasing every year with an aging demographic. Further, it is estimated that more than half (57%) of all people living with diabetes in Southeast Asia are undiagnosed.  Every day, you see many clients in your pharmacy seeking medicines and health advice. You have an opportunity to contribute to diabetes prevention, screening, and management for clients in your community. In this education module, you will learn how to identify risk factors for diabetes, counsel clients, recommend strategies and products for managing diabetes, and when to refer clients for care at the health facility.  Start the module now and take this first step to help your community prevent and manage diabetes. Click, Swipe, and learn! | <https://idf.org/our-network/regions-members/western-pacific/members/100-cambodia.html>  <https://www.who.int/diabetes/country-profiles/khm_en.pdf> |
| - 1. Diabetes types, risk factors, symptoms | Diabetes mellitus is a group of diseases related to the body’s ability to regulate blood glucose levels. There are three types of diabetes that will be discussed in this module: type 1 diabetes, type 2 diabetes, and gestational diabetes.  **WHAT IS INSULIN?**  Insulin is a peptide hormone made and secreted by cells in the pancreas. Without insulin, the body is unable to use and store sugar efficiently, leading to high blood glucose levels and further health serious health consequences such as hypertension and cardiovascular disease, blindness, kidney failure, and lower limb amputation.  **TYPE 1 DIABETES**  In type 1 diabetes, an autoimmune reaction causes the body to attack the cells in the pancreas which are responsible for making insulin. Because of this, people with type 1 diabetes are either unable to make insulin at all or enough of it. People with type 1 diabetes need to take insulin every day to live.  **TYPE 2 DIABETES**  Type 2 diabetes affects the most people (around 90% of people with diabetes have type 2) and is often acquired in adulthood, although adolescents are increasingly being diagnosed due to higher levels of child obesity.  Type 2 diabetes occurs when a person becomes insulin resistant, meaning the body no longer responds properly to insulin. In type 2 diabetes, consistently high blood glucose levels (i.e., due to high sugar diets and lack of exercise), encourages more insulin production. For many, the body gets overwhelmed, produces less and less insulin, and is no longer able to produce enough insulin in response to high blood glucose levels.  **GESTATIONAL DIABETES**  When a pregnant person is diagnosed with diabetes for the first time during pregnancy (gestation), this is referred to as gestational diabetes. Having gestational diabetes and high blood glucose during pregnancy is related to many serious health complications for the mother and child. Complications associated with gestational diabetes include: higher chances of C-section, early (preterm) birth, still-birth, serious breathing difficulties for the infant, and higher likelihood of acquiring type 2 diabetes for both the mother and infant.  As a pharmacist, it is important to ensure that pregnant clients are aware of the risks of gestational diabetes and are consistently monitoring their blood glucose levels, since many women do not recognize symptoms of gestational diabetes. You should also encourage clients to monitor their weight during pregnancy and flag drastic weight increases.  **Knowledge check: Match the description to the type of diabetes**   - - - 1. **This type of diabetes occurs when the body can’t respond properly to insulin (Type 2 diabetes)**       2. **This type of diabetes occurs when the body can’t make insulin (Type 1 diabetes)**       3. **This type of diabetes occurs during pregnancy (gestational diabetes)**   Understand the risk factors and symptoms of each type of diabetes and help your clients identify or prevent diabetes.   \|  \| **Risk factors** \| **Symptoms** \| \| --- \| --- \| --- \| \| **Type 1 Diabetes** \| - Having a family member with diabetes - Viral infections \| - Abnormal thirst and dry mouth - Sudden weight loss - Frequent urination - Lack of energy, tiredness - Constant hunger - Blurred vision - Bedwetting \| \| **Type 2 Diabetes** \| - Family history of diabetes - Being overweight - Having an unhealthy diet - Physical inactivity - Increasing age - High blood pressure - Ethnicity - Impaired glucose tolerance (IGT)* - History of gestational diabetes and poor nutrition during pregnancy \| - Excessive thirst and dry mouth - Frequent urination - Lack of energy, tiredness - Slow healing wounds - Recurrent infections in the skin - Blurred vision - Tingling or numbness in hands and feet. \| \| **Gestational Diabetes** \| - Being overweight - Physical inactivity - Previous gestational diabetes or prediabetes. - Polycystic ovary syndrome. - Diabetes in an immediate family member. - Previously delivering a baby weighing more than 9 pounds (4.1 kilograms). - Ethnicity \| - Many women do not observe obvious symptoms of gestational diabetes. Possible symptoms include frequent urination and increased thirst. \| | <https://www.idf.org/aboutdiabetes/type-1-diabetes.html> |
| - 1. Diabetes management and prevention | **Management and Prevention of Diabetes**  People living with type 1 diabetes and some people living with type 2 diabetes will need to take insulin to control their blood glucose levels. The type of insulin taken and when it is taken will be recommended by a physician, however, keeping track of blood glucose levels is important to inform insulin dosage. We will review self-monitoring of diabetes in depth in later sections of the course.  A healthy diet and exercise are vital components of diabetes management. Engaging in these lifestyle behaviours can prevent a person from acquiring type 2 diabetes or -disease progression which requires oral anti-diabetes medication (i.e., Metformin and Sulfonylureas, SGLT2s, DPP-IV inhibitors) and insulin. In addition to regular glucose monitoring, understanding what and when to eat as well as engaging in physical activity is also important in the management of type 1 diabetes, to control sugar levels in the blood.  You should recommend the following to your clients to help them manage diabetes:  Encourage clients to take their medication as prescribed by their doctor.   - Encourage clients to reduce the amount of calories eaten if they are overweight. - Replace saturated fats (i.e., cream, cheese, butter) with unsaturated fats (i.e., avocados, nuts, olive, vegetable oils) - Eat less high-glycemic foods (rice, bread, sweets) and eat more high-fiber (vegetable, fruit, whole grains) - Avoid using tobacco - Avoid drinking sugary drinks and excessive alcohol - Drink more water and less tea and coffee - Exercise for at least 30 minutes per day. Regular physical activity should include a mix of resistance training and cardio (i.e., running, swimming biking). - Manage stress with yoga, meditation and exercise |  |
| - 1. Diabetes and COVID-19 | Amidst the COVID-19 pandemic, people living with diabetes should take extra precaution to protect themselves. People with diabetes are both more susceptible to viral infection as well as experiencing severe illness requiring hospitalization and death if infected with COVID-19.  You would advise clients with diabetes to continue COVID-19 prevention practices such as wearing masks, socially distancing, washing and sanitizing hands frequently, avoid crowded spaces, and getting the COVID-19 vaccine if accessible. Also encourage clients to maintain good glycaemic control, by continuing to take their prescribed medication as well as testing their blood glucose regularly. If a client shares that they are experiencing COVID-19 symptoms or have had recent contact with someone confirmed to be infected with COVID-19, you encourage them to take a WHO-EUL rapid-antigen test and comply with local guidelines for confirmatory testing, self-isolation and reporting as recommended by the MOH. | <https://www.idf.org/aboutdiabetes/what-is-diabetes/covid-19-and-diabetes/1-covid-19-and-diabetes.html> |
| - 1. The Role of Pharmacists | As a pharmacist, you can play a significant role in diabetes prevention, screening, and management.  You can help inform clients of their risk for diabetes and encourage them to adopt health lifestyle behaviours for prevention. At the time of diagnosis, more than 50% of people with diabetes in Cambodia were already experiencing one or more diabetes-related complications. Identifying diabetes early among high-risk individuals, can potentially contribute to earlier diagnosis and prevention of severe diabetes complications.  According to the National Guidelines, at-risk clients who should be screened for diabetes include those who:   - Are overweight (BMI>23, waist circumference in men ≥85cm and in women≥80cm) - Have family history of diabetes - Have hypertension (BP>140/90), dyslipidaemia - Have a history of stroke or ischaemic heart disease - Women with a previous history of gestational diabetes - Women who have given birth to a large baby(>3500g) - Those who are over 35   Refer clients who are at-risk of diabetes and/or are experiencing symptoms of diabetes (outlined in the table above) and have not been diagnosed. Also refer clients already diagnosed with diabetes to the health facility if they report very high glucose levels. The typical target for blood glucose levels for those living with diabetes is 80-130mg/dL right before meals and 130-180mg/dL around 2 hours after meals (more on blood glucose monitoring in the next section).  If possible, find opportunities to collaborate with other health care providers on personalized management plans for clients with diabetes and engage in support such as keeping consistent stock of diabetes products such as diabetes medication and blood glucose monitoring devices and strips; supporting medication and insulin adherence; and monitoring physical activity, diet, and glucose levels.   \| **Interactive Case Study 1:** (with pictures and using a survey monkey tool built into the CPD so that participants can receive feedback on their response immediately):  A middle-aged client approaches a pharmacist and explains he has hypertension, complains of a wound that won’t heal, tiredness and blurred vision. What would the pharmacist’s next steps be (multiple correct answers)?   1. **Ask the client if they have ever received a diagnosis of diabetes.** 2. Tell the client to go home and rest, they are probably just fatigued. 3. **Tell the client to go to the health facility to test for diabetes** 4. Dispense the client antibiotics and tell them to report back if their symptoms clear up. \| \| --- \| | National Guidelines. <https://niph.org.kh/niph/uploads/library/pdf/GL236_DM_guideline_moh_en.pdf>  <https://www.ncbi.nlm.nih.gov/pmc/articles/PMC5774315/>  IPF webinar:  <https://www.youtube.com/watch?v=p2boZw7XVgg> |
| Section 2: Blood glucose monitoring | | |
| Section 2: Blood glucose monitoring | Clients with diabetes should be regularly assessing their blood glucose levels.  If clients are taking insulin, it is important that they monitor their sugar levels multiple times a day to ensure that they are receiving the right dosage of insulin and to prevent blood glucose from spiking or dropping too low.. In addition, glucometers are convenient tools which can inform clients with diabetes about how specific foods, physical activities, and medication change their blood glucose.  **Using a blood glucose monitoring device and strip**  If you stock blood glucose meters in your pharmacy, be sure to read the manufacturer’s instructions so that you can properly counsel clients or help them perform a measurement.  In general, most glucose monitoring products include a lancet, which is used to prick the finger, a test strip which the client would place the drop of blood on, and a meter, which provides a reading of the blood glucose level measurement. Remind your clients to wash and dry their hands thoroughly before conducting a test and to have a diary and pen ready to record the results. Having a record of daily test results can educate clients about their diabetes journey and inform health care providers about the progress and appropriateness of the treatment strategy. There are also blood glucose meters available that connect to applications on mobile phones with logbook functionality.  [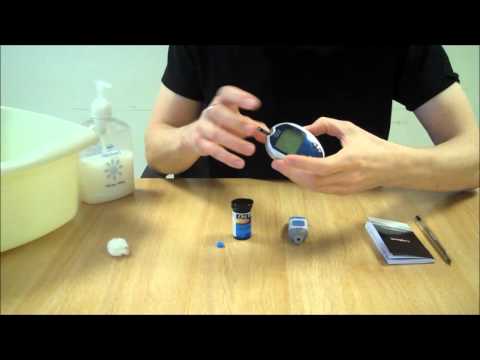](https://www.youtube.com/embed/rMMpeLLgdgY?feature=oembed)  **Frequency of glucose monitoring and interpreting results**  For clients with type 1 diabetes, it is recommended that they test at least 4 times a day. Typically, tests should be taken at minimum as often as insulin is taken, before a meal, and 2 hours after a meal. Encourage clients to speak with their health care provider about how best to incorporate self-monitoring in their diabetes management plan.  When using blood glucose monitoring devices, remind your clients about the glucose level targets are below:   \| Before meals: \| 80 – 130mg/dl \| \| --- \| --- \| \| 2 hours after meals: \| 130 – 180mg/dl \|   Remind clients to tell their physician if their results are consistently high. Depending on the client’s history, medication or insulin may be considered in the treatment plan by their physician. If results are higher than the recommended targets, encourage clients to engage in lifestyle choices to bring those levels down, such as exercise and a healthy diet.  If possible, integrate the following into our CPD:  <https://guidelines.diabetes.ca/self-management/smbg-tool-pwd#:~:text=your%20healthcare%20provider.-,Check%20your%20blood%20sugar%20at%20least%20as%20often%20as%20you,least%204%20times%20a%20day>.  **HbA1c Testing**  Every 6 months, clients living with diabetes should also get a hemoglobin A1c (HbA1c) test. In contrast to blood glucose monitoring products which measures the concentration of glucose in the blood at the moment the finger is pricked, HbA1c tests assess average blood levels over 2-3 months, by measuring the percentage of red blood cells with sugar-coated hemoglobin (protein in RBCs which glucose attaches to). The target for diabetes patients is a level of less than 7%. Refer clients living with diabetes to the health facility if they have not received an HbA1c test in last 6 months.  Topics:   - Importance of accurate blood glucose monitoring (to reliably monitor this key parameter of diabetes management and inform appropriate therapy decisions) and factors that can affect accuracy - ISO 15197:2013 accuracy requirements for blood glucose monitoring products. What does this mean and how do I know whether a product has ISO 15197 certification   **What is Accuracy**  **Accuracy** refers to the closeness of a measured value to a standard or known value.  Analytical Accuracy = Analytical accuracy of a blood glucose meter is a measure of how closely bG measurements match the true blood glucose  Clinical Accuracy = estimates whether it is still possible to make a correct therapeutic decision based on the result obtained  **Why is Accuracy important for BGM:**  Accurate blood glucose results are essential for reliable self monitoring of blood glucose by people living with diabetes and for taking the right treatment decision (e.g. dosing of insulin)  In addition self monitoring of blood glucose is the most practical method for detection of asymptomatic hypoglycaemia  **What is ISO and why is it relevant for BGM**  ISO is an abbreviation for International Organisation for Standardisation  ISO is an independent and non-governmental organisation.  The ISO 15197 is an international standard, which describes minimum  performance requirements (analytical and clinical accuracy) for blood glucose monitoring systems used by lay persons.    The evaluation also includes the testing of 23 potential interfering substances eg: acetaminophen, ascorbic acid etc and the testing of the interference of haematocrit on the accuracy of the bgm.  ISO 15197 applies to blood glucose monitoring systems that measure glucose concentrations in capillary blood.  It is not for blood glucose monitoring in professional use and for continuous blood glucose monitoring.  The ISO 15197:2013 is applicable to manufacturers of such systems and those other organizations (e.g. regulatory authorities and conformity assessment bodies) having the responsibility for assessing the performance of these systems.  The ISO standard 15197 is applicable for the pre market evaluation of SMBG systems. | -National Guidelines  -2021 SwipeRx KAP Survey Results  -ISO standard 15197:2013 descriptions of minimum accuracy requirements for BGM systems  IDF materials [here](https://www.idf.org/aboutdiabetes/what-is-diabetes/covid-19-and-diabetes/1-covid-19-and-diabetes.html)  IDF guideline: Self-Monitoring of Blood Glucose in Non-Insulin Treated Type 2 Diabetes  <https://www.youtube.com/watch?v=NUskHl7APHE>  https://labwrite.ncsu.edu/Experimental%20Design/accuracyprecision.htm  <https://www.ncbi.nlm.nih.gov/pmc/articles/PMC3320815/>  <https://www.iso.org/standard/54976.html> |
| Section 3: Diabetes management counseling | When counselling clients living with diabetes, it may be helpful to remember the the “4Ms”: Medication, Monitoring, Movement & Meals for managing their diabetes.   - - - - **Medication**: review a client’s medication plan if they are taking anti-diabetes medication (i.e., metformin, sulphonylureas) or insulin. Encourage clients to take the dosages at frequencies according to how their physician recommended and to test their HbA1c levels every 6 months.       - **Monitoring**: share about the benefits of blood glucose monitoring, especially if clients are taking insulin to treat their diabetes. If clients have never used blood glucose monitoring tools, show them how to conduct a test.       - **Movement & Meals:** as previously mentioned, physical activity and healthy eating is important for blood glucose level management and weight control. If clients are overweight (BMI>23, waist circumference in men ≥85cm and in women≥80cm), encourage them to lose weight and recommend healthy meal options and tips to motivate them to move around more and sit down less.  \| **Interactive Case Study 2:** creative team to create graphics to go alongside tips based on the 4Ms.  Remember the 4Ms when counselling diabetes: Medication, Monitoring, Movement, and Meals. Here are some helpful tips that you can share with your clients for each of the M’s:  **MEDICATION:** Keep organized! You can suggest to clients that they use tools like pillboxes or scheduled alarm clocks so that they find ways to remember the medication they need to take.  [picture of pillbox and calendar]  **MONITORING:** Make sure clients don’t forget to track the results of their glucose tests and encourage them to write in a diary of their lifestyle progress, including details like weight progress, emotions, workout plans, etc.  [picture of a log notebook or mobile tracking app]  **MOVEMENT:** Here are some small lifestyle changes you can suggest to your clients with diabetes on how they can be more active and less sedentary:  [picture of stairs] – take the stairs instead of the elevator!  [picture of someone riding a bicycle] – ride a bike to get around, instead of taking the car or motorcycle  [picture of someone running in the park] – enjoy the park while going on a morning run  **MEALS:** Encourage clients to eat healthy food – to AVOID saturated fats and high-sugar foods and drinks and REPLACE them with unsaturated fats and fibre foods like vegetables, whole grains, and fruit.  MEAL GAME: FRIEND OR FOE (using Survey Monkey): Identify whether each picture below is appropriate for individuals with diabetes to eat (friend) or should be avoided by people with diabetes (foe).   1. [picture of grilled –not fried local fish] 🡪 FRIEND 2. [picture of white rice with fried meat] 🡪 FOE; instead try brown rice or baked orange flesh sweet potato with lean, low-fat meat 3. [picture of bubble tea] 🡪 FOE; instead try fresh fruit 4. [picture of fried chicken] 🡪 FOE; instead try grilled chicken breast 5. [picture of butter] 🡪 FOE; instead try olive oil 6. [grilled vegetables] 🡪 FRIEND 7. [xxxx] 🡪 FRIEND \| \| --- \|   **Signs and symptoms of hypoglycemia**    Also ensure that clients with diabetes understand the signs and symptoms of hypoglycemia, or low blood glucose, such as: irregular or quickened heartbeat, fatigue, pale skin, sweating, hunger, irritability, tingling in the lips, tongue, or cheeks, irritability, and feeling lightheaded or dizzy, and anxiety. More severe hypoglycemia may result in fainting, seizures, and blurred vision.  Clients with diabetes should take extra care to continuously monitor blood glucose so that levels do not dip too low, especially for those taking insulin and sulphonylureas (anti-diabetes medication that stimulates the pancreas to create more insulin). If clients experience these symptoms, advise them to take glucose tablets, drink juice or soft drinks, or eat candy. If symptoms persist, clients should seek medical attention.  **Revisiting COVID-19 and diabetes management**  Given their increased risk of infection and serious illness from COVID-19, when speaking to clients living with diabetes, it is important to remind them to practice prevention measures such as wearing masks, sanitizing and washing hands with soap thoroughly, and social distancing. If clients with diabetes come in contact with someone who has tested positive for COVID-19, or if they are experiencing symptoms, you can advise them to screen themselves using COVID-19 rapid antigen tests found in pharmacies and health facilities. Clients can also stay vigilant by controlling their glucose levels to limit the risk of complications if they contract COVID-19.  **Measuring blood pressure**  Since people with diabetes are at a higher risk of hypertension and cardiovascular disease, these clients should be measuring their blood pressure frequently – at least once every 3 months. If their blood pressure is consistently high (greater than 130 mmHg/80 mmHg), refer them to a health facility to ensure possible hypertension is treated early to prevent microvascular and cardiovascular diseases. You can also advise them to limit salt intake – try swapping soy sauce, fish sauce, and MSG out for seasoning with lemon, peppers, and herbs.  **Empower clients to stick with their management plan**  Behaviour change is not always easy for clients. As a key health care provider in your community, practice compassionate care and encourage clients to adhere to diabetes management practices such as taking anti-diabetes medication and/or insulin, maintaining a healthy lifestyle.  Help pharmacy clients commit to recommended diabetes management practices at home:   - - - - Encourage them to stick to their plan for their family and for themself, so that they can be healthy enough to enjoy life to the fullest.       - Talk through diet and exercise goals and plans with clients.       - Advise clients to record glucose measurement results in a diary or digital device.       - If you see some clients on a regular basis, praise them if they are able to keep relatively normal blood glucose levels and stick to their plan. If clients do not see any or worse progress over time, provide encouragement and coach them by suggesting ways they might overcome barriers.       - If clients come with family, engage with them to help support the client through their journey with diabetes.   Answers to frequently asked questions by pharmacy clients   \| **Frequently Asked Questions by Pharmacy Clients based on SwipeRx Survey Findings** \| **Answer** \| **Source** \| \| --- \| --- \| --- \| \| Are there any side effects associated with using diabetes meds for a long time? \| Short-term side effects may be experienced while using diabetes medications, depending on types or classes of drugs you are taking. If you have questions or are experiencing longer-term side effects, seek advice from your primary provider. \| <https://www.diabetes.org/healthy-living/medication-treatments/oral-medication/what-are-my-options> \| \| What lifestyle changes can I purse to manage diabetes? \| Regular physical activity along with healthy diet can help you manage diabetes. \| <https://www.niddk.nih.gov/health-information/diabetes/overview/diet-eating-physical-activity.#whatFood> \| \| Can I change my diabetes drug without prescription? \| You should NOT change your prescription without your doctor’s approval. \| <https://www.niddk.nih.gov/health-information/diabetes/overview/insulin-medicines-treatments#injectable> \| \| What is the normal or acceptable BG level in diabetes patient? What is acceptable BG level after meal? \| It is recommended to have 80-130mg/dl before meals, and 130-180mg/dl 2 hours after meals. \| <https://www.mayoclinic.org/diseases-conditions/diabetes/diagnosis-treatment/drc-20371451> \|   **Interactive quiz: How much do you know about diabetes terminologies and concepts?**  Series of Images/Cards with a True/False to be provided:   1. Insulin is the hormone required to process sugar in the body **(TRUE).** 2. Hyperglycemia is the dangerous state of low blood glucose **(FALSE).** 3. Blood glucose meter is a machine using test strips to estimate blood glucose level **(TRUE)** 4. Pancreas is a body organ that is responsible for insulin production **(TRUE)** 5. Type 2 diabetes is caused by the complete absence of insulin **(FALSE)**  \| Interactive Case Study 3: (with pictures and using a survey monkey tool built into the CPD so that participants can receive feedback on their response immediately)  A client with diabetes asks the pharmacist for advice because she is worried that she may have been exposed to someone with COVID-19 in her office last week. How should the pharmacist counsel in this case (multiple correct options possible):   1. Diabetes clients have less risk of contracting COVID-19 2. **Unmanaged diabetes increases the risk of experiencing serious health issues associated with COVID-19; to prevent the risk of complications from COVID-19, individuals with COVID-10 should manage blood glucose levels with routine monitoring, medication adherence, exercise movement and diet meals ( suggest to stick with the 4M’s)** 3. **Recommend use of a rapid antigen test to screen for COVID-19** 4. **Recommend vigilant use of COVID-19 prevention practices including handwashing with soap, mask wearing avoidance of crowds and ventilation of indoor spaces** 5. Recommend that the client go straight to the health facility 6. **Recommend that the client go to a health facility if the rapid antigen test is positive or if COVID-19 symptoms persist** \| \| --- \|  \| Interactive Case Study 4: (with pictures and using a survey monkey tool built into the CPD so that participants can receive feedback on their response immediately)  A client with Type 2 diabetes is treated with oral anti-diabetes medication. What advice should the pharmacist give the client?   1. **Take the medication exactly as recommended by the physician for your treatment plan.** 2. **Educate the client about symptoms of hypoglycemia and what to do if they feel symptoms, especially if there they are taking sulphonylureas or insulin.** 3. **Educate the client about the benefits of blood glucose monitoring** 4. **Encourage clients to maintain a healthy weight with daily exercise of at least 30 minutes and a good diet** 5. **Tell the client that they should be measuring their blood pressure at least once every 3 months.** 6. Tell the client that a benefit of the monitoring blood glucose levels regularly is that they can reduce their visits to the health facility to only once a year for general check-up. \| \| --- \| | <https://www.who.int/emergencies/diseases/novel-coronavirus-2019/media-resources/science-in-5/episode-46---diabetes-covid-19> |

**Pre-/Post-module Questions:**

1. Which of the following are the recommended times to test blood glucose levels for someone with diabetes?

a) Before breakfast ( fasting)

b) Two hours after a meal

c) Changing or adjusting insulin or medication

d) Before bed

**e) All of the above**

- **Answer:** ideally, diabetic clients on treatment should measure blood glucose levels after each meal and before bed. You can encourage your client to seek guidance from her/his doctor regarding recommended frequency, but generally clients diagnosed with diabetes should measure blood glucose levels several times a day. In addition, measurement is recommended when there are changes to a person’s routine while travelling; when experiencing either high or low blood glucose symptoms; when pregnant or trying to get pregnant; before and after surgical procedures.

2. What blood glucose level is recommended for diabetes clients two hours after eating?

a) 60–80 milligrams per deciliter

b) 80–120 mg/dL

c) 120–160 mg/dL

**d) Less than 180 mg/dL**

e) More than 190 mg/dL

3. Which of the following is *not* recommended for appropriate blood glucose monitoring

a) Clients (and pharmacist or someone else assisting) should wash hands with soap prior to measuring blood glucose levels

**b) Clients should lie down when measuring or having blood glucose measured**

c) Client or pharmacist should insert test strip into test meter before collecting blood sample

d) Client or pharmacist should prick the side of fingertip with the lancet provided with the test kit

e) Client should touch test strip to blood and wait a few seconds for glucose reading

- **Answer:** blood glucose can be measured while clients are sitting, standing or lying down –though lying down is neither recommended nor needed for an accurate measurement

4. Which of the following lifestyle practices can help clients with diabetes manage blood glucose levels?

a) Exercise at least 30 minutes/day

b) Drink more water and less tea, coffee, soda and alcohol

c) Eat less high-glycemic foods (bread, rice, sweets) and eat more high-fiber (vegetable, fruit)

d) Manage stress with yoga, meditation and exercise

e) **All of the above**

5. Which of the following is true about diabetes and COVID-19?

a) There is no connection between diabetes and COVID-19 health risks

b) Clients with diabetes are less likely to experience severe health consequences, if infected with COVID-19

**c) Clients with poorly controlled diabetes are more likely to experience severe health consequences, if infected with COVID-19**

d) Clients with diabetes should only use PCR testing for COVID-19, as rapid antigen tests are not suitable for diabetes clients

e) Clients with diabetes do not need to worry about wearing a mask or practicing other recommended COVID-19 prevention practices

- **Answer:** Individuals with diabetes (or other non-communicable diseases including cancer and hypertension) are more likely to experience serious consequences requiring hospitalization and may face increased risk of COVID-19 related mortality compared to individuals without diabetes co-morbidity. For this reason it is important for pharmacists to encourage clients with diabetes to practice vigilant COVID-19 prevention practices (mask wearing, avoiding crowds, ventilating home/work spaces) and screen for COVID-19 using a rapid antigen test if they experience COVID-19 symptoms or are exposed to someone with COVID-19. This is also why it is so important for pharmacists to encourage clients to improve blood glucose management, so that they can prevent the risk of serious COVID-19 consequences, should they become infected in future.

6. What do the ISO 15197;2013 standards mean for people living with diabetes

a) These are the standards for minimum exercise requirements for people living with diabetes

**b) These are the standards for BGM system accuracy, ensuring reliable results**

c) These are the standards for dietary requirements for people living with diabetes

d) These are the standards for how to interpret blood glucose levels

e) All of the above

7. Which of the following is *not* a benefit of diabetes clients owning their own BGM meter and test strips?

a) Ability to self-monitor glucose levels

**b) Ability to avoid going to the health facility**

c) Ability to conveniently measure glucose levels, any time of day or location

d) Ability to frequently measure glucose levels

e) Ability to assess impact of diet, sleep or exercise on glucose levels

- **Answer:** even clients who self-monitor glucose levels regularly need to visit a health facility for diabetes medical assessments as recommended by their doctor. If your client has concerns about visiting a health facility, you can explain that the benefits of seeing a doctor at a health facility far outweigh any hassle or costs.

8 (Cambodia only). When counselling diabetes clients, which of the following should you should cover:

a) Medication: importance of adhering to doctor recommended insulin or oral anti-diabetes medication

b) Regular blood glucose monitoring

c) Meals: how to manage glucose levels with good diet

d) Movement: specific ways they can sit less and move more in the week ahead

**e) All of the above**

9. A client comes to ask your advice on how to use a blood glucose meter. You notice the test strips they are using have expired. What advice/ recommendations would you give to ensure they perform an accurate blood glucose test

1. In order to ensure the bg result is accurate, the client would need to obtain a new vial of test strips. Using expired test strips is not appropriate and may provide an inaccurate result.
2. Before testing the blood glucose, client should ensure their hands are washed with soap and water, and dried.
3. Client should ensure that they close the test strip vial, and keep the vial stored at room temperature. The test strips should be stored in the original container.

10 (Cambodia only): True or False: individuals with diabetes are at high risk of hypertension and cardiovascular disease

**True:** diabetes can damage arteries which can increase the risk of hypertension and cardiovascular disease. Managing diabetes with regular glucose monitoring, treatment adherence and regular exercise can also reduce the risk that individuals with diabetes also suffer from cardiovascular disease.

11. Why are accurate BGM results important:

1. To prevent diabetes
2. To reliably monitor this important parameter in diabetes management
3. To prevent hypertension
4. To inform correct therapy decisions
5. **b & d**

12. Several factors can affect the accuracy of BGM results. Which one is not relevant?

1. Temperature and humidity
2. Application of an insufficient volume of blood
3. **Testing after a meal**
4. Use of expired or damaged test strips
5. Interfering substances like acetaminophen ( paracetamol)

13 (Cambodia only). What is HbA1c?

a. Test for vitamin A deficiency

b. Test for severe hypertension

c. Test for diabetic retinopathy

**d. Test to measure the amount of blood glucose attached to hemoglobin (the red blood cells that carry oxygen from your lungs to the rest of your body**

e. Test for gestational diabetes

14. Which of the following is a risk factor for Type 2 diabetes?

a. Family history of diabetes or gestational diabetes

b. Overweight

c. Age over 35 years

d. Women who have given birth to a large baby (>3500g)

e. **All of the above**

**Supplementary File 3.** Survey responses^a^ by urban and rural classification

| **Survey question** | **Cambodia** | | | **Viet Nam** | | |
| --- | --- | --- | --- | --- | --- | --- |
|  | **Urban, n (%)** | **Rural, n (%)** | **𝝌2** **test**  **(p-value)** | **Urban, n (%)** | **Rural, n (%)** | **𝝌2 test (p-value)** |
| Which of the following do you sell in your pharmacy? (select all that apply) |  |  |  |  |  |  |
| Blood glucose test strips | 196 (72) | 72 (64) | 1.97 (p=0.161) | 257 (92) | 72 (74) | 22.18  (p<0.001) |
| Blood glucose meters | 203 (74) | 64 (57) | 10.70 (p=0.001) | 209 (75) | 34 (35) | 50.77  (p<0.001) |
| Insulin | 60 (22) | 11 (10) | 7.72 (p=0.005) | 127 (46) | 22 (23) | 15.89  (p<0.001) |
| Oral anti-diabetic medication | 247 (90) | 104 (93) | 0.71 (p=0.400) | 231 (83) | 77 (79) | 0.680 (p=0.411) |
| Have you received training or education on diabetes-related care or products within the last 3 years? If so, please indicate the topics (select all that apply) |  |  |  |  |  |  |
| Diabetes symptoms and risk factors | 109 (40) | 50 (45) | 0.78 (p=0.378) | 155 (56) | 85 (88) | 31.71 (p<0.001) |
| Insulin | 30 (11) | 8 (7) | 1.30 (p=0.255) | 83 (30) | 22 (23) | 1.84 (p=0.175) |
| Oral anti-diabetes medication | 103 (38) | 26 (23) | 7.39 (p=0.007) | 139 (50) | 34 (35) | 6.47  (p=0.011) |
| Blood glucose monitoring products | 64 (23) | 13 (12) | 6.87 (p=0.009) | 129 (46) | 33 (34) | 4.49  (p=0.034) |
| Who provided the diabetes-related education? (select all that apply) |  |  |  |  |  |  |
| Ministry of Health | 75 (27) | 32 (29) | 0.06 (p=0.811) | 75 (27) | 22 (23) | 0.69 (p=0.405) |
| Pharmacy Council/Association | 60 (22) | 25 (22) | 0.01 (p=0.927) | 67 (24) | 20 (21) | 0.49 (p=0.484) |
| Pharmaceutical company | 100 (36) | 26 (23) | 6.38  (p=0.012) | 132 (48) | 58 (60) | 4.36  (p=0.037) |
|  | **Urban, n (range)** | **Rural, n  (range)** | **U test**  **(p-value)** | **Urban, n (range)** | **Rural, n (range)** | **U test (p-value)** |
| Among all of your clients seeking diabetes products (any type), approximately how many of those clients purchase blood glucose test strips per week? | 3 (2–6) | 3 (1–6) | 5,250 (p=0.848) | 8 (4–24)* | 8 (3–12) | 12,884 (p=0.006) |
| Among all of your clients seeking diabetes products (any type), approximately how many of those clients purchase blood glucose test meters per week? | 2 (1–4) | 1 (1–2) | 2,858 (p=0.015) | 3 (1–7) | 1 (0–5) | 5,025 (p=0.049) |
| Among all of your clients seeking diabetes products (any type), approximately how many of those clients purchase insulin products per week? | 3 (2–8) | 1.5 (1–3) | 148 (p=0.026) | 4 (1–12.5) | 2 (0–8) | 4,553 (p=0.040) |

^a^Selected survey questions only, *means higher mean rank

**Supplementary File 4.** Survey respondents knowledge of frequency of blood glucose level checks

| **Survey question, n (%)** | **Cambodia**  **(N=382)** | **Viet Nam**  **(N=375)** |
| --- | --- | --- |
| For a client diagnosed with diabetes prescribed multiple daily doses of insulin, how frequently should they check their blood glucose level? |  |  |
| Once a month | 38 (10) | 114 (30) |
| Once a week | 83 (22) | 67 (18) |
| Once a day | 124 (32) | 130 (35) |
| Several times in the day | 71 (19) | 51 (14) |
| Don’t know | 66 (17) | 13 (3) |

**Supplementary File 5.** Questions asked by diabetes-related clients, as reported by survey respondents

| **Question** | **Category** |
| --- | --- |
| Which BGM product is good to use, affordable and with accuracy tested? | BGM related products and their effectiveness |
| How often should I monitor my Blood glucose levels? | Testing frequency |
| Can I eat fruit? What food do I have to be on diet? | Diet and exercise |
| Treatment and disease condition | BGM related products and their effectiveness |
| Glucose level and product usage | Blood glucose level |
| Is Diabetes Curable? What products should they use when they are diabetes? | Diabetes symptoms, causes, etc. |
| If he has high blood glucose when he has an ulcer, can he be a diabetes? | Diabetes symptoms, causes, etc. |
| How much glucose level does it become overhead? | Blood glucose level |
| What food do I have to be on diet for? If my blood glucose become normal, can I stop taking anti-diabetes medication? | Diet and exercise |
| Do you have BGM and how do I use it? | BGM related products and their effectiveness |
| Is the disease cured? Is the treatment take much time? What are the benefits of using the anti-diabetes medication? | BGM related products and their effectiveness |
| Asked to help you check your Blood glucose level, what should I do? | Blood glucose level |
| Food diet | Diet and exercise |
| Is Diabetes Curable? Which health facilities are the best for diabetes treatment? Can I buy medicine in advance? Does diabetes is genetic? Does diabetes infect when we share dish? | Multiple BGM questions |
| What are the symptoms of diabetes? Do I need to take anti-diabetes for life? | Diabetes symptoms, causes, etc. |
| What foods should we eat for diabetes to prevent high blood glucose? Asked to seeking a glucose test? | Diet and exercise |
| What drug should I take if the blood glucose still high? | BGM related products and their effectiveness |
| Asked about glucose level | Blood glucose level |
| What food should we are on diet? Can I stop taking medicine when the blood glucose low | Diet and exercise |
| How long do I take anti-diabetes medication? Is diabetes curable? and how do I monitor diabetes? | Use of BGM products |
| How much sugar should I take per day? | Blood glucose level |
| How do I know being diabetes? | Diabetes symptoms, causes, etc. |
| Foods can spike the glucose level? | Diet and exercise |
| Whether or not my pharmacy sell Diabetes drug? | Other |
| Symptoms related diabetes that make them feel uncomfortable | Diabetes symptoms, causes, etc. |
| How is his blood glucose? | Blood glucose level |
| Is diabetes a hereditary disease? | Diabetes symptoms, causes, etc. |
| Is dizzy kind of low glucose symptoms? | Diabetes symptoms, causes, etc. |
| What food should he be on diet for? What medication should he take to lower his blood glucose faster? | Diet and exercise |
| How many times a day will you use BGM test kit? | Testing frequency |
| Feel tired and exhausted | Other |
| What food can they eat? Can they buy antidiabetic drugs without consulting a doctor? | Diet and exercise |
| Diabetes is curable? How long patients we to take? Will it cause to renal failure as being heard? | Diabetes symptoms, causes, etc. |
| Diabetes is curable? | Diabetes symptoms, causes, etc. |
| about lifestyle. prevention, and how to be healthy | Diet and exercise |
| Recommended food for diabetes patients | Diet and exercise |
| Whether or not selling BGM monitoring machine? | Other |
| How to use, its quality & and how easy to find test strip for longer use? | Use of BGM products |
| Foods to avoid? is it curable? Need to take the drugs for life? It causes renal failure? Injecting Insulin means the situation is in serious case? | Multiple BGM questions |
| Foods to avoid? what are the side effects? | Diet and exercise |
| The glucose level which able to indicate the Diabetes | Blood glucose level |
| 1. Can he/she stop using the drug? 2. Which BGM Machine/strip is good in terms of quality and affordable price? Esp. test strip which often out of stock! | BGM related products and their effectiveness |
| Asking/discuss about the suspected diabetes symptoms: fatigue, sweat, and blurry eye..! | Diabetes symptoms, causes, etc. |
| Food to avoid or to reduce, the complication of drug | Diet and exercise |
| Normal Blood glucose level for diabetes? If good, is means they can stop using the drug? | Blood glucose level |
| How to manage/control the glucose level ? | Blood glucose level |
| The drug will cause the renal failure? | BGM related products and their effectiveness |
| Taking vitamin C or Multivitamin in the effervescent form could spike glucose level? | BGM related products and their effectiveness |
| Which BMG Product is best? Most accurate result? | BGM related products and their effectiveness |
| The proper diet, effect & side effect of drug, cost of drug and BGM | BGM related products and their effectiveness |
| Foods to avoid and reduce the amount, the level of glucose (normal, and over?) | Diet and exercise |
| Posology of the drug | BGM related products and their effectiveness |
| Symptoms of diabetes? if there any curable drugs? | Diabetes symptoms, causes, etc. |
| When can I stop injecting insulin? And what are the side effect of insulin? | Use of BGM products |
| Range of glucose level (normal, high?) | Blood glucose level |
| What are the differences of diabetes and the high level of glucose? | Blood glucose level |
| How to control and manage the glucose level ? | Blood glucose level |
| Whether taking the med for life? | Use of BGM products |
| Can they switch the brand name? | Use of BGM products |
| Food to avoid, whether or not can switch the brand name, the level of glucose which indicate the diabetes | Diet and exercise |
| How much for BGM product? | BGM related products and their effectiveness |
| Where to get the proper treatment and care? | Testing Facility |
| Advise on how to take care health (general), esp. with diabetes? | Diet and exercise |
| Glucose level which indicates/ diagnosed diabetes ? | Blood glucose level |
| How many time to test glucose in one day ? | Testing frequency |
| The high level of glucose and complication | Blood glucose level |
| Posology of med, how often to use BGM monitoring product? | Use of BGM products |
| Brand Name, cost, and quality | BGM related products and their effectiveness |
| The quality | BGM related products and their effectiveness |
| The level of blood sugar -normal-, duration of using & food | Blood glucose level |
| The suspected symptoms and Other to be diagnosed as diabetes | Other |
| How often he/she need to check their A1c? | Testing frequency |
| Food to avoid? when to take drug for best efficacy? | Diet and exercise |
| Need to take med daily? food to eat? | Diet and exercise |
| Which glucose level is in normal or high range? How to manage? | Blood glucose level |
| When is the best time to test their blood test? | Testing frequency |
| How often to test glucose level per day? | Testing frequency |
| How to use BGM product and side effect of each? | Use of BGM products |
| Which BGM machine is the best one in terms of quality ? | BGM related products and their effectiveness |
| Why there is a fluctuation of blood glucose, up and down- even though same med | Blood glucose level |
| Food to avoid? Why there is a numbness of the leg? | Diet and exercise |
| When is the best time to take drug? | Testing frequency |
| Whether or not can take other supplement? | BGM related products and their effectiveness |
| Is it curable? | Diabetes symptoms, causes, etc. |
| What is the level range of glucose indicates diabetes? | BGM related products and their effectiveness |
| When is the best time to test the blood? | Testing frequency |
| Even though taking the drug, why the glucose level is not better? | BGM related products and their effectiveness |
| Should they stop using diabetes med once the level of glucose dropped drastically? |  |
| Is it curable? | Diabetes symptoms, causes, etc. |
| Is it curable? | Diabetes symptoms, causes, etc. |
| Most of the customers asked when they were able to stop using med if he is maintaining well on glucose level? | BGM related products and their effectiveness |
| How to use BGM products, and proper diet | Use of BGM products |
| If the glucose level is dropping to the normal range, can he/she stop using/taking med? | Blood glucose level |
| Symptoms of high glucose level, and low level | Blood glucose level |
| Why taking med daily and regularly, but the glucose level is still the same? | BGM related products and their effectiveness |
| Which drug is better? | BGM related products and their effectiveness |
| Which med is good in terms of quality ? | BGM related products and their effectiveness |
| Diet and uses of med | Diet and exercise |
| Why eating lots of food but still feeling fatigue and no energy! | Diet and exercise |
| When was he/she able to stop using med? | BGM related products and their effectiveness |
| Which med (group or name) is good for his/her condition/ glucose level? | BGM related products and their effectiveness |
| What are the normal or high glucose range? | Blood glucose level |
| Diet? Is it curable? | Diet and exercise |
| Is it curable? | Diabetes symptoms, causes, etc. |
| Asking about the active ingredients of each med | BGM related products and their effectiveness |
| Suspected symptoms for the high glucose level which can potentially cause diabetes | Diabetes symptoms, causes, etc. |
| Is it curable? | Diabetes symptoms, causes, etc. |
| Diet for diabetes patient, food/supplement could help my conditions? | Diet and exercise |
| Is it curable if he/she takes the med for years? | Diabetes symptoms, causes, etc. |
| Which of BGM machine is the best? | BGM related products and their effectiveness |
| Diet | Diet and exercise |
| When to switch to another med if the current one is not helpful enough? | BGM related products and their effectiveness |
| Manufacturer of the each BGM products, and about the quality | BGM related products and their effectiveness |
| Med uses | Use of BGM products |
| Types of med, diet, cost | Use of BGM products |
| Rank of glucose level- normal or high- | Blood glucose level |
| Health complication of diabetes & what to avoid when having Diabetes | Diabetes symptoms, causes, etc. |
| Why the med is not working? the glucose level is still high | BGM related products and their effectiveness |
| Health complications of diabetes | Diabetes symptoms, causes, etc. |
| Should it be checked daily or regularly? | Testing frequency |
| Consumers often ask about the symptoms, diet esp., the energy beverage. For BGM Products, the quality and the price & how to use | Multiple BGM questions |
| The products can and cannot be used for diabetes products | BGM related products and their effectiveness |
| High level of glucose mean diabetes? Can I use the med for treatment? | Blood glucose level |
| Level rank of glucose for diabetes diagnosis | Blood glucose level |
| The reliability of the BGM machine, test strip for each product? (the brand name, etc) | BGM related products and their effectiveness |
| Non-pharmacological treatment to lower down the glucose level | Other |
| What causes diabetes? When do we start treatment? What are the treatments? | Diabetes symptoms, causes, etc. |
| What medications should be prescribed for diabetics? Which type of medicine is good? What is the level of sugar considered high? | Multiple BGM questions |
| Is it curable? | Diabetes symptoms, causes, etc. |
| Seeking for recommending on milk for diabetes patients | Diet and exercise |
| Cause of diabetes, and price of each BGM product | Multiple BGM questions |
| Finding the med they takes daily such as Clamid, diamicron, glucophage, diabetmin, etc and asking on how to use! | BGM related products and their effectiveness |
| Diet | Diet and exercise |
| Symptoms of diabetes | Diabetes symptoms, causes, etc. |
| 1. Level of glucose, whether or not need to take med 2. What are the most fastest and effective ways to lower down the glucose level?  3. Symptoms of high glucose level  4. Which of diabetes are the best ones? So on so forth ... | Blood glucose level |
| Which med should be taken?  Whether test blood sugar daily? | BGM related products and their effectiveness |
| Asking for BGM, and the price of each product | BGM related products and their effectiveness |
| Should they take diabetes medicine if the glucose level is 115? | Blood glucose level |
| High glucose level means I need to take diabetes med? | Blood glucose level |
| The level of glucose rank- normal & high or low? | Blood glucose level |
| Curable? Diet? Lab test and symptoms? | Diabetes symptoms, causes, etc. |
| The normal rank of glucose level | Blood glucose level |
| Suspected symptoms of diabetes | Diabetes symptoms, causes, etc. |
| Food to avoid? | Diet and exercise |
| Always telling about symptoms like blurred vision, fatigue, tingling on his food, can't sleep | Diabetes symptoms, causes, etc. |
| Symptoms related question: can't sleep, very tired, frequent urination | Diabetes symptoms, causes, etc. |
| Food to avoid? Fruit to eat? | Diet and exercise |
| Seeking for BGM machine for diabetes patient | BGM related products and their effectiveness |
| Food to avoid? | Diet and exercise |
| Food to eat/ and medicine uses! | Diet and exercise |
| Alcohol is allowed? food to avoid? | Diet and exercise |
| The glucose rank- normal and high which indicated to diabetes? | Blood glucose level |
| Symptoms | Diabetes symptoms, causes, etc. |
| Consulting about suspected condition, and asking to confirm whether or not he/she is having diabetes? How can he/she monitor their blood glucose? | Multiple BGM questions |
| Taking diabetes drug is just a short term usage or for life? | BGM related products and their effectiveness |
| Price, quality, and duration for using | BGM related products and their effectiveness |
| Which BGM Products have the good quality with the affordable price? | BGM related products and their effectiveness |
| Price | BGM related products and their effectiveness |
| Should I take med daily and regularly? | BGM related products and their effectiveness |
| Do they need to take the Diabetes med for life? if so, what are the health complicationd for long term use? | BGM related products and their effectiveness |
| How can I lower down my blood sugar level? | Blood glucose level |
| Asking about med | BGM related products and their effectiveness |
| How long we need to wait to see the result after we takes the med? | BGM related products and their effectiveness |
| If I got a slightly high blood sugar level, do I need to take diabetes med? How should I do next if my glucose level is till high esp. during the pandemic? | Blood glucose level |
| Even though taking med, the glucose level is still high!? Why? | BGM related products and their effectiveness |
| 1- How can the disease get better? 2- Can his disease be cured? 3- When you are very tired, what should you do? 4- Is this device good to use? 5- How is it used? Can it be used for a long time? Is the result correct? | Multiple BGM questions |
| Which product is good, durable and affordable? | BGM related products and their effectiveness |
| When stop taking medication, does your blood sugar level rise again? | BGM related products and their effectiveness |
| Hyperglycaemia, how to use a glucose meter, how and how to take a hypoglycaemic drug | Use of BGM products |
| What are the health complications of diabetes & side effects of diabetes drugs? | BGM related products and their effectiveness |
| What is normal blood sugar level? What is the level considered as high? | Blood glucose level |
| Ask for diabetic medicines | BGM related products and their effectiveness |
| Glucose level ranked for normal- high or low? | Blood glucose level |
| Asking about diabetes drug | BGM related products and their effectiveness |
| BGM meter | Use of BGM products |
| glucose level before and after meal (normal-high-low) | Blood glucose level |
| Most are related to the complications of diabetes, the side effects of taking medicines and long term use of BGM meters | Diabetes symptoms, causes, etc. |
| Glucose level (normal-high-low)-- food to avoid- how to test blood sugar level? | Blood glucose level |
| Asking for BGM Product | Use of BGM products |
| Food to avoid | Diet and exercise |
| Why the blood sugar is still high? | Blood glucose level |
| Which diabetes medicines have less side effects and tolerance for them? Good in quality with affordable price? | BGM related products and their effectiveness |
| Symptoms, about food to eat, monitoring glucose | Multiple BGM questions |
| Do you have glucose testing machine? | Use of BGM products |
| This diabetes med is good? what should I do when I got fatigue/very tired? | BGM related products and their effectiveness |
| The health complications of diabetes to my health | Diabetes symptoms, causes, etc. |
| Health complications of living with diabetes | Diabetes symptoms, causes, etc. |
| Taking med for quite long, but the glucose level is still high and not good! | BGM related products and their effectiveness |
| Why taking diabetes drugs cause lose weight? | BGM related products and their effectiveness |
| Is diabetes genetic? | Diabetes symptoms, causes, etc. |
| The signs of hyperglycaemia and hypoglycaemia and seeking for advice to choose the BGM machine | Diabetes symptoms, causes, etc. |
| The posologies of meds | BGM related products and their effectiveness |
| Asking about which brands/ products have the higher quality? | BGM related products and their effectiveness |
| Is diabetes curable? Does he/she need to take the med regularly and forever? What food to avoid? | Diabetes symptoms, causes, etc. |
| Symptoms | Diabetes symptoms, causes, etc. |
| Posologies of the medicines | BGM related products and their effectiveness |
| What are the glucose level which indicate diabetes? | Diabetes symptoms, causes, etc. |
| Is diabetes curable? What are the foods to avoid? | Diabetes symptoms, causes, etc. |
| What are the long terms side effect of diabetes use? | BGM related products and their effectiveness |
| What are the complications of diabetes? And where is the reliable healthcare facility which I can go to? | Diabetes symptoms, causes, etc. |
| What is the result of my blood glucose test? | Blood glucose level |
| How to use the BGM monitoring machine? | Use of BGM products |
| What are foods to avoid and what are allowed? | Diet and exercise |
| When is the best time to take/ use the BGM product? | Testing frequency |
| 1. What are the diabetes type 2 symptoms? 2. What are the causes of diabetes?  3.Which BGM products have the most quality, accuracy, efficacy and safety? | Multiple BGM questions |
| About medicine | BGM related products and their effectiveness |
| Symptoms and the glucose level which indicates the signs of diabetes | Blood glucose level |
| The side effects and the most effective drugs | BGM related products and their effectiveness |
| Asked about daily dietary | Diet and exercise |
| Asked about Blood glucose level | Blood glucose level |
| Asked about taking anti-diabetes medication | Use of BGM products |
| Asked about type of anti-diabetes medication | Use of BGM products |
| Asked about BGM test strips and BGM meter | Use of BGM products |
| Can we stop taking anti-diabetes medication? What are disadvantage of taking anti-diabetes medication too long? | BGM related products and their effectiveness |
| Asked whether if Blood glucose level, on anti-diabetes medication, what kind of food should be on diet and what exercise for diabetes patients should I take? | Multiple BGM questions |
| Asked about the symptoms of diabetes | Diabetes symptoms, causes, etc. |
| Asked about why the blood glucose go up and down | Blood glucose level |
| Regarding to the usage of anti-diabetes medication unless the blood glucose go down, should I continue or take more dose of this medication? | Use of BGM products |
| How many times a day should I take this type of anti-diabetes medicine? | Testing frequency |
| Drug information | BGM related products and their effectiveness |
| Asked about symptoms of diabetes? | Diabetes symptoms, causes, etc. |
| How to keep your blood glucose stability? How to keep energy while Blood glucose levels go down? | Blood glucose level |
| What food should be on diet? | Diet and exercise |
| Can I take diabetes medication without measuring my blood glucose? What are the side effects of anti-diabetes medication? | Use of BGM products |
| Why do I feel exhausted after taking anti-diabetes medication? | BGM related products and their effectiveness |
| What Blood glucose level do consider to be diabetes? | Blood glucose level |
| Asked about anti-diabetes medication usage, and Why does blood glucose still go up whether if taking medicine? | BGM related products and their effectiveness |
| What Blood glucose level do consider to be diabetes? | Blood glucose level |
| Asked about symptoms of diabetes | Diabetes symptoms, causes, etc. |
| What food should be on diet? Can I be cure?, How to use BGM and insulin? | Diet and exercise |
| What food should be on diet? | Diet and exercise |
| Asked about anti-diabetes medication | BGM related products and their effectiveness |
| Daily dietary | Diet and exercise |
| What food should be on diet? Can I take some exercise? What drugs are good? | Diet and exercise |
| Change anti-diabetes medication | BGM related products and their effectiveness |
| Do you sell anti-diabetes medication?, When do we you take insulin? What is BGM quality? | Use of BGM products |
| Treatment | Testing Facility |
| Diabetes conditions | Diabetes symptoms, causes, etc. |
| Can diabetes be cured? | Diabetes symptoms, causes, etc. |
| How do I know whether I have got diabetes? What are the symptoms of diabetes?, What factors cause diabetes? | Diabetes symptoms, causes, etc. |
| What kind of anti-diabetes medication do I take? Symptoms of diabetes? | BGM related products and their effectiveness |
| What should I do when the blood glucose goes up? | Blood glucose level |
| Diabetes condition | Diabetes symptoms, causes, etc. |
| Side effects, diabetes symptoms | Diabetes symptoms, causes, etc. |
| Asked about BGM and what blood glucose is normal and daily dietary? | Multiple BGM questions |
| What Blood glucose level should be diabetes? | Blood glucose level |
| Asked about anti-diabetes medication and dietary | BGM related products and their effectiveness |
| Why do I frequently feel dizzy? | Diabetes symptoms, causes, etc. |
| Asked about serving food, symptoms, Blood glucose level and frequently pee | Diet and exercise |
| What are the side effects of taking prolonged anti-diabetes medication? | Diabetes symptoms, causes, etc. |
| Doctor prescription | Other |
| Seeking blood test and anti-diabetes medication | Use of BGM products |
| What are the general symptoms of diabetes? | Diabetes symptoms, causes, etc. |
| Diet some food and taking exercise | Diet and exercise |
| I feel dizzy, tired, sweaty, my hands and feet shaking. | Diabetes symptoms, causes, etc. |
| Do you sell anti-diabetes medication? | BGM related products and their effectiveness |
| What Blood glucose level should be diabetes? | Blood glucose level |
| Symptoms | Diabetes symptoms, causes, etc. |
| Symptoms | Diabetes symptoms, causes, etc. |
| Blood glucose level | Blood glucose level |
| What food should be on diet and asked about diabetes symptoms | Diet and exercise |
| Type of anti-diabetes medication | BGM related products and their effectiveness |
| What food should be on diet and asked about diabetes symptoms | Diet and exercise |
| Asked about cause of diabetes, prevention and treatment and BGM use | Multiple BGM questions |
| What food should be on diet and asked about diabetes symptoms | Diet and exercise |
| Asked what nutrient supplement should take and especially fresh milk and food that diabetes patients can serve | Diet and exercise |
| Normal blood glucose | Blood glucose level |
| Asked about diabetes symptoms and seek for BGM test | Diabetes symptoms, causes, etc. |
| Should I take some medicine if there is no prescription? | BGM related products and their effectiveness |
| How frequently should I check blood glucose per month? | Testing frequency |
| Can I stop taking anti-diabetes medication? | BGM related products and their effectiveness |
| Can I change the higher level of anti-diabetes medication? | BGM related products and their effectiveness |
| Asked for anti-diabetes and its usage | BGM related products and their effectiveness |
| Side effects of diabetes, the usage of anti-diabetes medication, how to live with diabetes, check-up blood glucose and health | Diabetes symptoms, causes, etc. |
| Asked about diabetes symptoms, the food should be on diet and what should we do daily? | Diabetes symptoms, causes, etc. |
| Blood glucose didn't go down | Blood glucose level |
| Normal blood glucose | Blood glucose level |
| With high blood glucose what drug should we take? | BGM related products and their effectiveness |
| How do we test our blood glucose in the right way? | Testing frequency |
| What food should we are on a diet for diabetes patients? | Diet and exercise |
| What food should we are on a diet for diabetes patients? | Diet and exercise |
| How much blood glucose we should take medicine? | BGM related products and their effectiveness |
| How accurate are BGM tests? | BGM related products and their effectiveness |
| Blood glucose still stand even though we take anti-diabetes medication | BGM related products and their effectiveness |
| Asked about anti-diabetes medication usage | Use of BGM products |
| Can diabetes be cured? | Diabetes symptoms, causes, etc. |
| Can we take some juice drinks? | Diet and exercise |

**Supplementary File 6**. Factors considered by survey respondents when recommending blood glucose monitoring products in A) Cambodia and B) Viet Nam


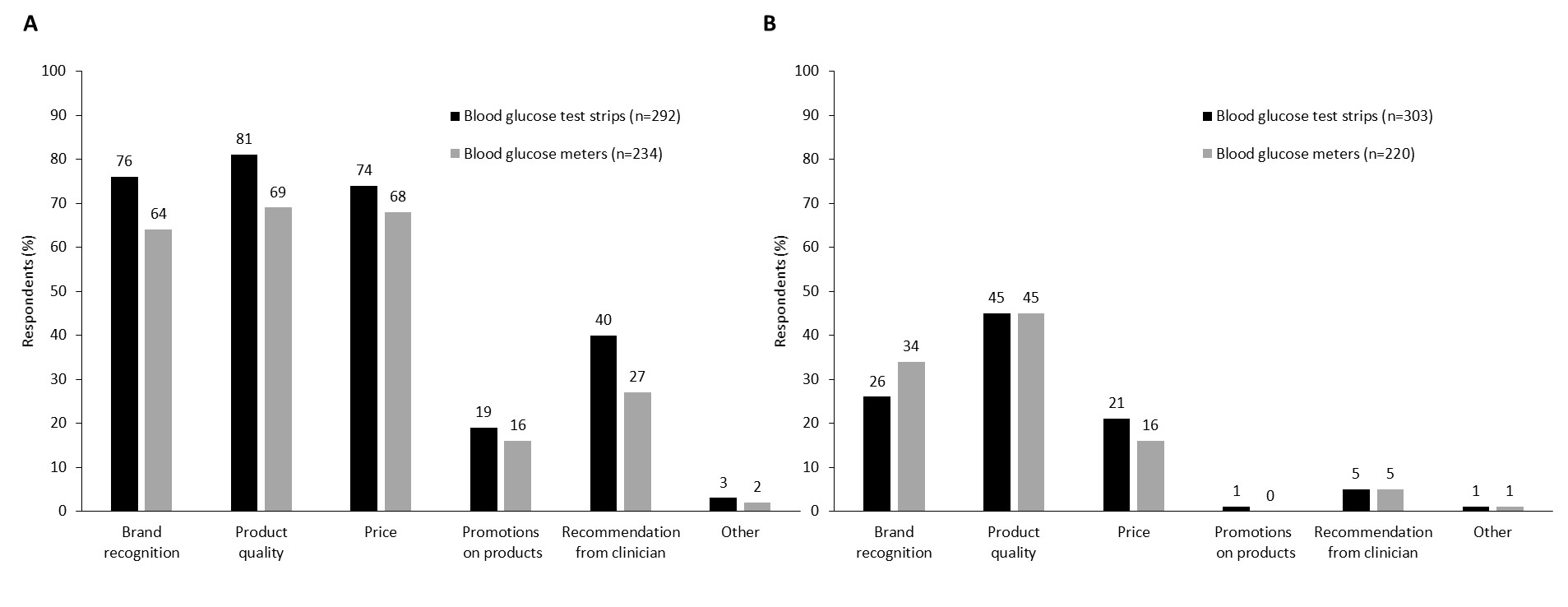

Supplement: Supplementary file 1 — Supplementary Material 1 [file 12909_2023_4449_MOESM1_ESM.docx]
